# Overexpressed pseudogenes, DUXAP8 and DUXAP9, promote growth of renal cell carcinoma and serve as unfavorable prognostic biomarkers

**DOI:** 10.18632/aging.102152

**Published:** 2019-08-13

**Authors:** Jing Chen, Weiyang Lou, Bisha Ding, Xian Wang

**Affiliations:** 1 Department of Medical Oncology, Sir Run Run Shaw Hospital, Medical School of Zhejiang University, Zhejiang Province, Hangzhou 313100, China; 2 First Affiliated Hospital of Jiaxing University, Zhejiang Province, Jiaxing 314000, China; 3 Department of Surgery, First Affiliated Hospital, College of Medicine, Zhejiang University, Hangzhou 313100, China

**Keywords:** pseudogene, kidney cancer, renal cell carcinoma (RCC), DUXAP8, DUXAP9

## Abstract

Background: Growing studies have reported that pseudogenes play key roles in multiple human cancers. However, expression and roles of pseudogenes in renal cell carcinoma remains absent.

Results: 31 upregulated and 16 downregulated pseudogenes were screened. Higher expression of DUXAP8 and DUXAP9 indicated poorer prognosis of kidney cancer. 33 and 5 miRNAs were predicted to potentially binding to DUXAP8 and DUXAP9, respectively. miR-29c-3p was identified as the most potential binding miRNAs of DUXAP8 and DUXAP9 based on expression, survival and correlation analyses. 254 target genes of miR-29c-3p were forecast. 47 hub genes with node degree >= 10 were identified. Subsequent analysis for the top 10 hub genes demonstrated that COL1A1 and COL1A2 may be two functional targets of DUXAP8 and DUXAP9. Expression of DUXAP8, DUXAP9, COL1A1 and COL1A2 were significantly increased in cancer samples compared to normal controls while miR-29c-3p expression was decreased. Luciferase reporter assay revealed that miR-29c-3p could directly bind to DUXAP8, DUXAP9, COL1A1 and COL1A2. Functional experiments showed that DUXAP8 and DUXAP9 enhanced but miR-29c-3p weakened growth of renal cell carcinoma.

Conclusions: In conclusion, upregulated DUXAP8 and DUXAP9 promote growth of renal cell carcinoma and serve as two promising prognostic biomarkers.

Methods: Dysregulated pseudogenes were obtained by dreamBase and GEPIA. The binding miRNAs of pseudogene and targets of miRNA were predicted using starBase and miRNet. Kaplan-Meier plotter was utilized to perform survival analysis, and Enrichr database was introduced to conduct functional enrichment analysis. Hub genes were identified through STRING and Cytoscape. qRT-PCR, luciferase reporter assay, cell counting assay and colony formation assay were performed to validate *in silico* analytic results.

## INTRODUCTION

Kidney malignancy is the twelfth most common cancer all over the whole world [1]. Renal cell carcinoma is the most common type of primary kidney malignancy and accounts for 90%–95% of all kidney cancer cases [2]. Among all subtypes of renal cell carcinoma (RCC), clear cell renal cell carcinoma (ccRCC) is the most common one, approximately accounting for 75% of all RCC cases [3]. In addition to ccRCC, there are also other subtypes, such as chromophobe renal cell carcinoma (chRCC) and papillary renal cell carcinoma (pRCC). Over the past few decades, great improvements have been achieved in the diagnosis, therapy and prognosis of RCC. However, the patients’ prognosis remains unsatisfactory, partially owing to the high rate of cancer recurrence after undergoing surgical resection [4]. Thus, it is necessary to unlock the molecular mechanisms of RCC onset and progression and develop novel effective diagnostic, therapeutic and prognostic biomarkers for RCC.

Pseudogenes, genomic DNA sequences similar to normal genes, are regarded as defunct relatives of functional genes [5, 6]. Pseudogenes are generally divided into two main types: processed (or retrotransposed) pseudogene and non-processed (or duplicated) pseudogenes [7–9]. After the first discovery of pseudogene structure in 5S DNA of *Xenopus laevis* by C. Jacq et al. in 1977 [10], increasing studies have demonstrated that dysregulation of pseudogenes plays key roles in development of human disorders and these deregulated pseudogenes may act as promising therapeutic targets for diseases [11, 12]. Recently, pseudogenes have emerged as critical regulators in the pathogenesis of cancer, including lung cancer [13], pancreatic cancer [14], hepatocellular carcinoma [9], brain glioma [15], colon cancer [16], gastric cancer [17, 18], breast cancer [19, 20] as well as kidney cancer [21].

Pseudogenes could serve as competing endogenous RNAs (ceRNAs) of their parental genes and other unrelated genes through competitively binding to shared miRNAs, thereby exerting a variety of biological functions [22]. For example, pseudogene OCT4-pg4 regulates OCT4 expression by competing for miR-145 in hepatocellular carcinoma [23]; pseudogene TUSC2P promotes TUSC2 function by binding multiple microRNAs [24]; a FTH1 gene-pseudogene-microRNA network modulates development of prostate cancer [25]. However, to the best of our knowledge, pseudogene-miRNA-gene network in renal cell carcinoma has not yet been fully elucidated. Exploration of functional pseudogenes associated with renal cell carcinoma and their corresponding ceRNA mechanism may provide novel insights for diagnosis, therapy and prognosis of renal cell carcinoma in the future.

In this study, we first screened differentially expressed pseudogenes in RCC by analyzing dreamBase and GEPIA. Next, we further found those prognosis- related pseudogenes among all the differentially expressed pseudogenes. Using starBase and miRNet databases, a pseudogene-miRNA-gene network was subsequently constructed to explore the underlying molecular mechanisms of key pseudogenes in RCC. Finally, a series of experiments, including qRT-PCR, luciferase reporter assay, cell counting assay and colony formation assay, were conducted to validate analytic results.

## RESULTS

### Screening dysregulated pseudogenes in human ccRCC

As mentioned above, ccRCC is the most common type of RCC. Therefore, differentially expressed pseudogenes in ccRCC was first obtained to explore the functional pseudogenes and excavate their potential mechanisms of action in RCC using human pseudogene database, namely dreamBase. Based on the cut-off criteria, a total of 399 dysregulated pseudogenes, containing 335 upregulated and 64 downregulated pseudogenes, were finally identified in human ccRCC (Supplementary Table 1). In order to confirm the preliminary results, we determined expression levels of these dysregulated pseudogenes in human ccRCC using another online database GEPIA. 31 out of 335 upregulated pseudogenes and 16 out of 64 downregulated pseudogenes were found to be consistent with the previous analytic results from dreamBase database as listed in Supplementary Table 2. The 47 differentially expressed pseudogenes were defined as potential pseudogenes and were selected for further analysis.

### Identification of DUXAP8 and DUXAP9 as two key pseudogenes in RCC

**Figure 1:**
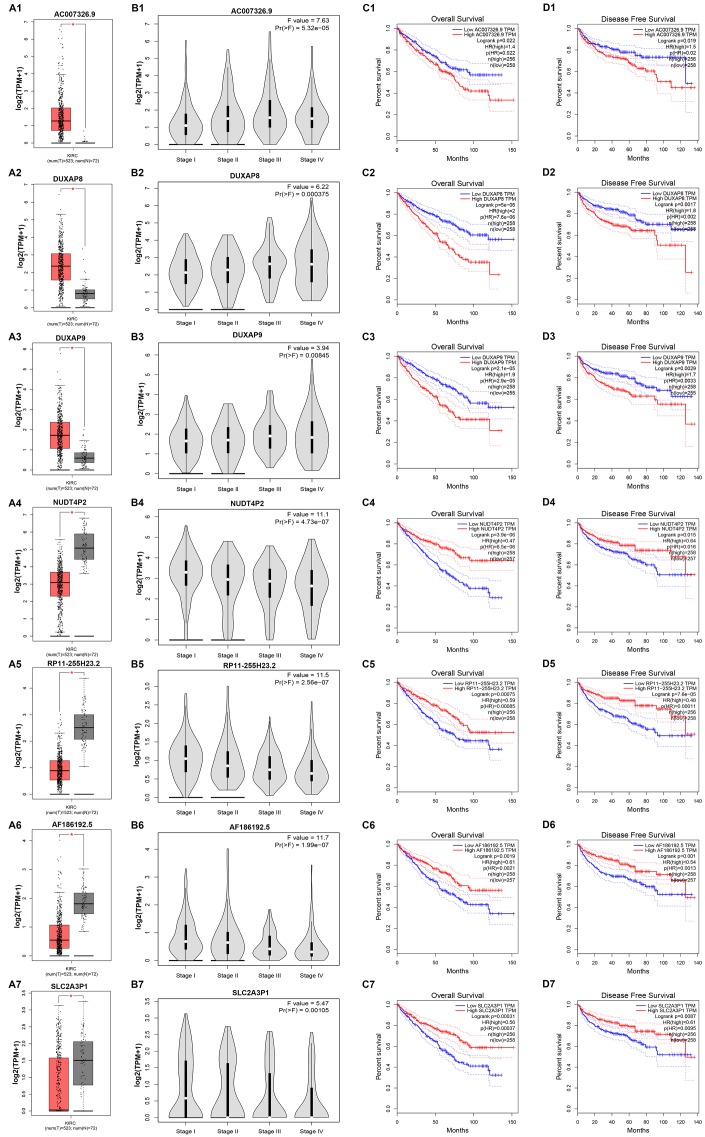
**Expression and prognostic values of 7 potential pseudogenes in clear cell renal cell carcinoma (ccRCC) determined by GEPIA database.** (**A1**) Expression of AC007326.9 in ccRCC compared with normal controls; (**A2**) expression of DUXAP8 in ccRCC compared with normal controls; (**A3**) expression of DUXAP9 in ccRCC compared with normal controls; (**A4**) expression of NUDT4P2 in ccRCC compared with normal controls; (**A5**) expression of RP11-255H23.2 in ccRCC compared with normal controls; (**A6**) expression of AF186192.5 in ccRCC compared with normal controls; (**A7**) expression of SLC2A3P1 in ccRCC compared with normal controls; (**B1**) expression of AC007326.9 among major stages in ccRCC; (**B2**) expression of DUXAP8 among major stages in ccRCC; (**B3**) expression of DUXAP9 among major stages in ccRCC; (**B4**) expression of NUDT4P2 among major stages in ccRCC; (**B5**) expression of RP11-255H23.2 among major stages in ccRCC; (**B6**) expression of AF186192.5 among major stages in ccRCC; (**B7**) expression of SLC2A3P1 among major stages in ccRCC; (**C1**) prognostic role (overall survival) of AC007326.9 in ccRCC; (**C2**) prognostic role (overall survival) of DUXAP8 in ccRCC; (**C3**) prognostic role (overall survival) of DUXAP9 in ccRCC; (**C4**) prognostic role (overall survival) of NUDT4P2 in ccRCC; (**C5**) prognostic role (overall survival) of RP11-255H23.2 in ccRCC; (**C6**) prognostic role (overall survival) of AF186192.5 in KIRC; (**C7**) prognostic role (overall survival) of SLC2A3P1 in ccRCC; (**D1**) prognostic role (disease free survival) of AC007326.9 in ccRCC; (**D2**) prognostic role (disease free survival) of DUXAP8 in ccRCC; (**D3**) prognostic role (disease free survival) of DUXAP9 in ccRCC; (**D4**) prognostic role (disease free survival) of NUDT4P2 in ccRCC; (**D5**) prognostic role (disease free survival) of RP11-255H23.2 in ccRCC; (**D6**) prognostic role (disease free survival) of AF186192.5 in ccRCC; (**D7**) prognostic role (disease free survival) of SLC2A3P1 in ccRCC. “*” represents P-value less than 0.05. Three horizontal lines in the box plot represent minimum, median and maximum, respectively.

**Figure 2:**
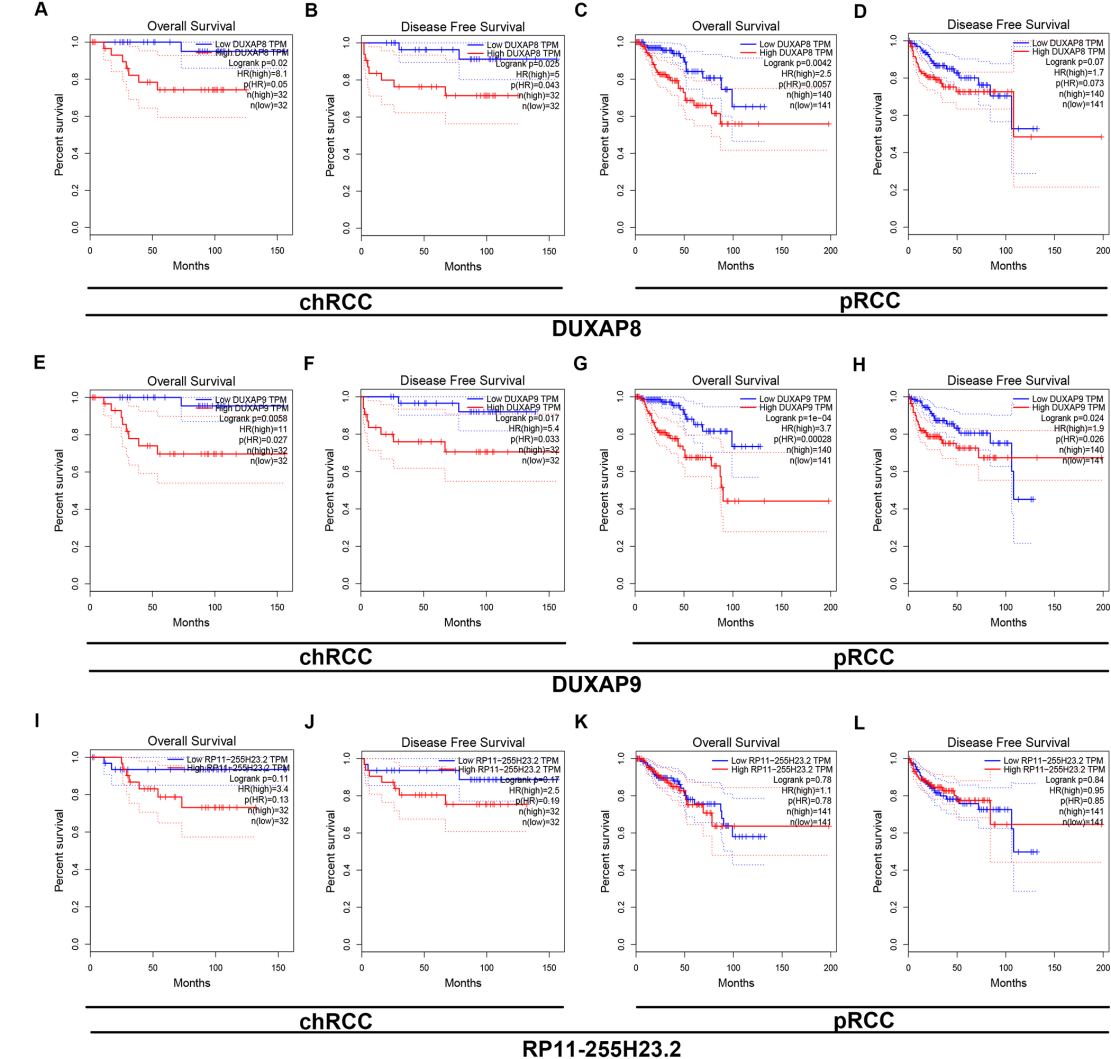
**Survival analysis of DUXAP8 and DUXAP9 in chRCC (chromophobe renal cell carcinoma) and pRCC (papillary renal cell carcinoma).** (**A**) Prognostic value (overall survival) of DUXAP8 in chRCC; (**B**) prognostic value (disease free survival) of DUXAP8 in chRCC; (**C**) prognostic value (overall survival) of DUXAP8 in pRCC; (**D**) prognostic value (disease free survival) of DUXAP8 in pRCC; (**E**) prognostic value (overall survival) of DUXAP9 in chRCC; (**F**) prognostic value (disease free survival) of DUXAP9 in chRCC; (**G**) prognostic value (overall survival) of DUXAP9 in pRCC; (**H**) prognostic value (disease free survival) of DUXAP9 in pRCCs.

Next, we determined the prognostic values of 47 potential pseudogenes in human ccRCC based on TCGA data using GEPIA database. Two prognostic indexes, consisting of overall survival (OS) and disease free survival (RFS), were included in this part. Among the 47 potential pseudogenes, only high expression of three upregulated pseudogenes (AC007326.9, DUXAP8 and DUXAP9) and four downregulated pseudogenes (NUDT4P2, RP11-255H23.2, AF186192.5 and SLC2A3P1) indicated poorer and better OS and RFS in patients with ccRCC, respectively (Supplementary Table 3). The expression levels of the 7 pseudogenes between cancer and normal samples were displayed in Figure 1A1-A7. Moreover, expression analysis among various major stages of the 7 pseudogenes demonstrated that all of them possessed statistical significance (Figure 1B1–B7). Figure 1C1–C7 and Figure 1D1–D7 revealed OS and RFS of the 7 pseudogenes, respectively. To discover the most potential functional pseudogenes in RCC, we performed survival analysis (OS and RFS) of the 7 pseudogenes in chRCC and pRCC. As listed in Table 1 and Figure 2, only chRCC and pRCC patients with higher expression of DUXAP8 and DUXAP9 predicted poorer prognosis. All these findings suggest that DUXAP8 and DUXAP9 may serve as two potential prognostic biomarkers in human RCC.

**Table 1:** Prognostic roles of 7 potential pseudogenes in chRCC and pRCC determined by GEPIA database.

**Pseudogene name**	**chRCC ^a^**		**pRCC ^b^**		**Effect^e^**
	**OS^c^**	**RFS^d^**	**OS^c^**	**RFS^d^**	
AC007326.9	No	No	No	No	No
**DUXAP8**	**Poor**	**Poor**	**Poor**	**No**	**Poor**
**DUXAP9**	**Poor**	**Poor**	**Poor**	**Poor**	**Poor**
NUDT4P2	No	No	No	No	No
RP11-255H23.2	No	No	No	No	No
AF186192.5	No	No	Good	Good	No
SLC2A3P1	No	No	No	No	No

^a^chRCC, chromophobe renal cell carcinoma;

^b^pRCC, papillary renal cell carcinoma;

^c^OS, Overall Survival;

^d^RFS, Disease Free Survival;

^e^Effect, total effect of individual pseudogene in chRCC and pRCC.

### Identification of miR-29c-3p as a potential binding miRNA of DUXAP8 and DUXAP9

**Figure 3:**
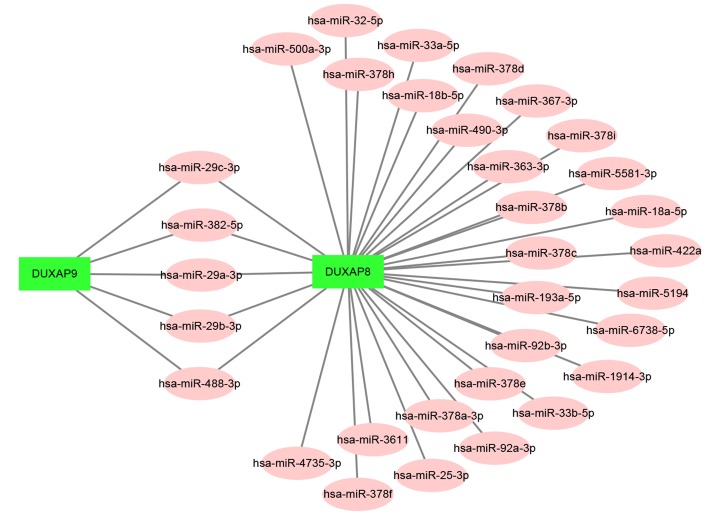
**Establishment of the potential DUXAP8/DUXAP9-miRNA regulatory network in renal cell carcinoma.**

**Figure 4:**
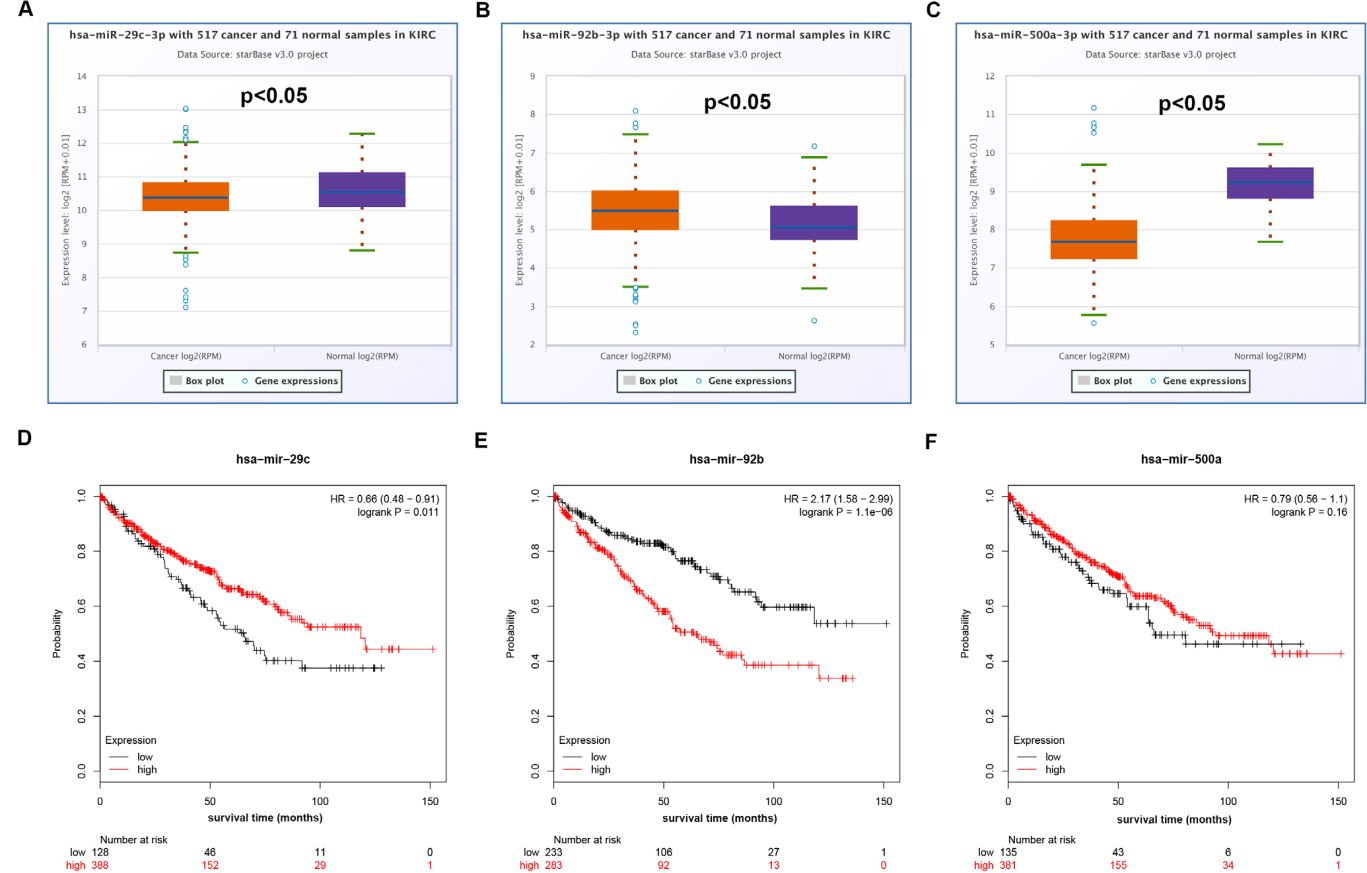
**Expression and survival analysis of miRNAs in clear cell renal cell carcinoma (ccRCC or KIRC).** (**A**) Box-whisker plot showed expression of hsa-miR-29c-3p in ccRCC compared with normal controls (three horizontal lines in the box plot represent minimum, median and maximum); (**B**) box-whisker plot showed expression of hsa-miR-92b-3p in ccRCC compared with normal controls (three horizontal lines in the box plot represent minimum, median and maximum); (**C**) box-whisker plot showed expression of hsa-miR-500a-3p in ccRCC compared with normal controls (three horizontal lines in the box plot represent minimum, median and maximum); (**D**) prognostic value of hsa-miR-29c-3p in ccRCC; (**E**) prognostic value of hsa-miR-92b-3p in ccRCC; (**F**) prognostic value of hsa-miR-500a-3p in ccRCC.

Increasing evidences have suggested that ceRNA hypothesis is an important regulatory mechanism for ncRNA, including lncRNA, circRNA as well as pseudogenes [22, 26]. Therefore, to uncover underlying mechanisms and functions of DUXAP8 and DUXAP9, we first predicted potential binding miRNAs by starBase database. Finally, 33 and 5 miRNAs were predicted to potentially bind to DUXAP8 and DUXAP9, respectively. For better visualization, DUXAP8/DUXAP9-miRNA network was established as presented in Figure 3. According to the ceRNA mechanism, there should be a negative correlation between miRNA expression and DRXAP8/9 expression. Thus, we determined the expression correlation using starBase database. As shown in Table 2, among these pseudogene-miRNA pairs, only four interactions (DUXAP8-miR-29c-3p, DUXAP8-miR-92b-3p, DUXAP8-miR-500a-3p and DUXAP9-miR-29c-3p) demonstrated significantly negative relationship in ccRCC. In the next step, we determined the expression and prognostic values of the three miRNAs (miR-29c-3p, miR-92b-3p and miR-500a-3p) in ccRCC. The results from expression analysis revealed that miR-29c-3p was significantly downregulated in ccRCC samples when compared with normal controls (Figure 4A). Figure 4B told us a marked upregulation of miR-92b-3p expression in cancer samples. We also found that miR-500a-3p expression in cancer tissues was significantly lower than that in normal tissues (Figure 4C). Survival analysis for the three miRNAs demonstrated that ccRCC patients with higher expression of miR-29c-3p and miR-92b-3p had better and worse prognosis as presented in Figure 4D and Figure 4E, respectively. Figure 4F showed that there was no significant survival difference between miR-500a-3p low expression group and miR-500a-3p high expression group. These findings demonstrated that miR-29c-3p expression was conversely correlated with DUXAP8 and DUXAP9 expression, and miR-29c-3p was significantly downregulated in ccRCC and the expression downregulation indicates a poor prognosis of ccRCC patients. As shown in Supplementary Figure 1A and Supplementary Figure 1B, miR-29c-3p was also significantly downregulated in chRCC and pRCC. Furthermore, we also found that pRCC patients with high expression of miR-29c-3p had a favorable prognosis (Supplementary Figure 1C). Taken together, miR-29c-3p may be a potential binding miRNA of DUXAP8 and DUXAP9 in renal cell carcinoma.

**Table 2:** Correlation of potential pseudogene and miRNA in clear cell renal cell carcinoma (ccRCC) determined by starBase version 3.

**Pseudogene**	**miRNA**	**R**	**P-value**
DUXAP8	hsa-miR-29b-3p	0.087	4.69E-02
**DUXAP8**	**hsa-miR-29c-3p**	**−0.158**	**2.97E-04**
DUXAP8	hsa-miR-29a-3p	0.06	1.75E-01
DUXAP8	hsa-miR-382-5p	0.239	3.87E-08
DUXAP8	hsa-miR-488-3p	−0.085	5.33E-02
DUXAP8	hsa-miR-4735-3p	0	1.00E+00
DUXAP8	hsa-miR-18a-5p	0.115	5.09E-02
DUXAP8	hsa-miR-18b-5p	−0.002	9.71E-01
DUXAP8	hsa-miR-5581-3p	0.008	8.99E-01
DUXAP8	hsa-miR-378b	−0.036	5.37E-01
DUXAP8	hsa-miR-378e	0.025	6.67E-01
DUXAP8	hsa-miR-378d	0.093	1.13E-01
DUXAP8	hsa-miR-378f	−0.002	9.72E-01
DUXAP8	hsa-miR-378i	0.006	9.22E-01
DUXAP8	hsa-miR-378a-3p	0.115	5.09E-02
DUXAP8	hsa-miR-422a	0	1.00E+00
DUXAP8	hsa-miR-378h	−0.057	3.34E-01
DUXAP8	hsa-miR-378c	0.095	1.06E-01
DUXAP8	hsa-miR-33b-5p	0.052	3.78E-01
DUXAP8	hsa-miR-33a-5p	0.084	1.52E-01
DUXAP8	hsa-miR-490-3p	0.156	8.06E-03
DUXAP8	hsa-miR-32-5p	0.101	8.68E-02
DUXAP8	hsa-miR-363-3p	−0.013	8.20E-01
DUXAP8	hsa-miR-367-3p	0	1.00E+00
DUXAP8	hsa-miR-25-3p	0.068	2.49E-01
DUXAP8	hsa-miR-92a-3p	0.23	8.07E-05
**DUXAP8**	**hsa-miR-92b-3p**	−**0.11**	**6.07E-02**
**DUXAP8**	**hsa-miR-500a-3p**	−**0.145**	**1.37E-02**
DUXAP8	hsa-miR-3611	0	9.95E-01
DUXAP8	hsa-miR-1914-3p	0.01	8.72E-01
DUXAP8	hsa-miR-5194	−0.016	7.82E-01
DUXAP8	hsa-miR-6738-5p	0	1.00E+00
DUXAP8	hsa-miR-193a-5p	0.164	5.10E-03
DUXAP9	hsa-miR-488-3p	−0.039	3.73E-01
DUXAP9	hsa-miR-29a-3p	0.066	1.36E-01
DUXAP9	hsa-miR-29b-3p	0.071	1.06E-01
**DUXAP9**	**hsa-miR-29c-3p**	−**0.142**	**1.24E-03**
DUXAP9	hsa-miR-382-5p	0.247	1.34E-08

### KEGG pathway enrichment, GO functional annotation, and PPI network analysis for miR-29c-3p target genes

**Figure 5:**
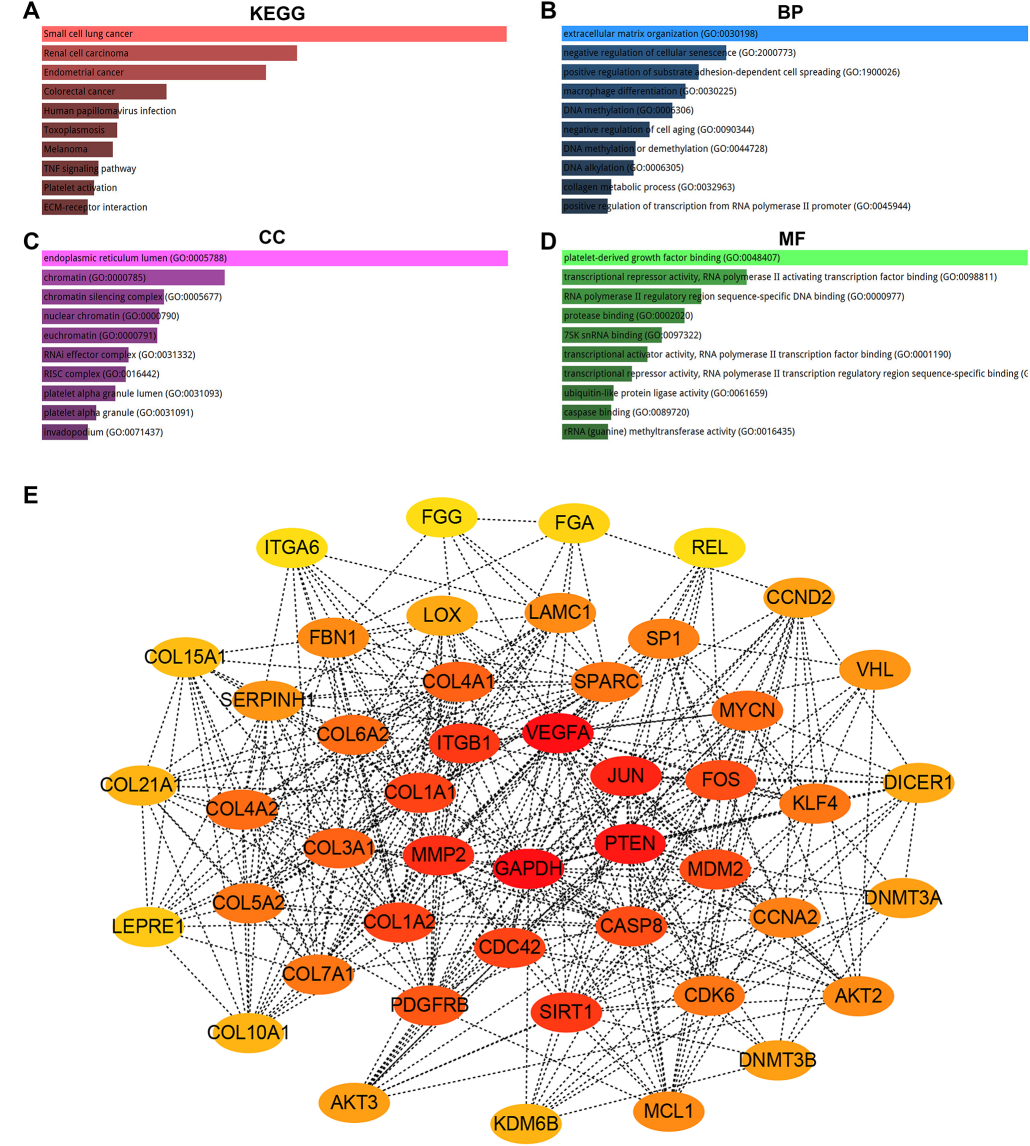
**KEGG pathway enrichment, GO functional annotation and PPI network analysis for target genes of hsa-miR-29c-3p.** (**A**) The top 10 enriched KEGG pathway items; (**B**) the top 10 enriched biological process (BP) items; (**C**) the top 10 enriched cellular component (CC) items; (**D**) the top 10 enriched molecular function (MF) items; (**E**) the top 47 hub genes (node degree >= 10) in PPT network of target genes.

For better understanding the potential functions of pseudogene DUXAP8 and DUXAP9, the target genes of miR-29c-3p were first predicted using a comprehensive tool, miRNet. As shown in Supplementary Table 4, a total of 254 potential target genes were obtained. Next, we mapped them into Enrichr database for KEGG pathway enrichment analysis and GO functional annotation. Three GO items (biological process, BP; cellular component, CC; molecular function, MF) were included in GO functional annotation. KEGG pathway analysis for these target genes demonstrated that they were significantly enriched in several human cancers and cancer-associated pathways, such as small cell lung cancer, renal cell carcinoma, endometrial cancer, colorectal cancer, melanoma, TNF signaling pathway and ECM-receptor interaction (Figure 5A). A variety of significant enriched GO terms were discovered and the top 10 enriched GO terms were shown in Figure 5B–5D, including extracellular matrix organization (GO:0030198), negative regulation of cellular senescence (GO:2000773) and positive regulation of substrate adhesion-dependent cell spreading (GO:1900026) in the BP category, endoplasmic reticulum lumen (GO:0005788), chromatin (GO:0000785) and chromatin silencing complex (GO:0005677) in the CC category, and platelet-derived growth factor binding (GO:0048407), transcriptional repressor activity, RNA polymerase II activating transcription factor binding (GO:0098811) and RNA polymerase II regulatory region sequence-specific DNA binding (GO:0000977) in the MF category. It has been widely acknowledged that genes exert their biological functions by interacting with each other instead of acting alone. Thus, we established the PPI network of aforementioned potential target genes of miR-29c-3p using STRING database. The result showed that many gene-gene interactions among these target genes were existed (Data were not shown). To obtain the hub genes, we re-entered the gene-gene interactions into Cytoscape software (version 3.6.0). The node degree of each target gene was calculated by CytoHubba plugin of Cytoscape software. For better visualization, the hub genes with node degree >= 10 were screened (Table 3) and a sub-PPI network were re-constructed as shown in Figure 5E.

**Table 3:** The hub genes in the PPI network (node degree >= 10).

**Gene name**	**Node degree**
GAPDH	46
VEGFA	46
PTEN	38
JUN	35
MMP2	31
SIRT1	28
ITGB1	28
CDC42	25
COL1A1	25
COL1A2	25
MDM2	24
CASP8	24
FOS	24
PDGFRB	23
COL3A1	22
COL4A1	22
MYCN	21
COL6A2	21
COL4A2	21
KLF4	20
COL7A1	20
SPARC	20
COL5A2	20
CDK6	20
SP1	19
CCNA2	19
FBN1	18
LAMC1	18
MCL1	18
AKT2	18
SERPINH1	17
VHL	17
AKT3	16
DNMT3A	16
DNMT3B	16
CCND2	16
LOX	15
DICER1	15
COL21A1	14
KDM6B	14
COL10A1	14
COL15A1	13
LEPRE1	12
FGA	11
REL	10
ITGA6	10
FGG	10

### COL1A1 and COL1A2 are identified as functional target genes of DUXAP8 and DUXAP9

**Figure 6:**
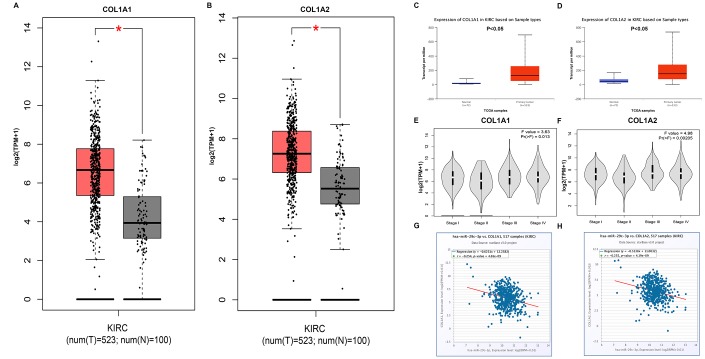
**COL1A1 and COL1A2 were identified as two potential target genes of DUXAP8 and DUXAP9.** (**A**) Box-whisker plot represented expression of COL1A1 in clear cell renal cell carcinoma (ccRCC or KIRC) compared with normal controls determined by GEPIA database; (**B**) Box-whisker plot represented expression of COL1A2 in ccRCC compared with normal controls determined by GEPIA database; (**C**) expression of COL1A1 in ccRCC compared with normal controls determined by UALCAN database; (**D**) expression of COL1A2 in ccRCC compared with normal controls determined by UALCAN database; (**E**) expression of COL1A1 among major stages in ccRCC determined by GEPIA database; (**F**) expression of COL1A2 among major stages in ccRCC determined by GEPIA database; (**G**) correlation analysis between has-miR-29c-3p and COL1A1 in ccRCC; (**H**) correlation analysis between has-miR-29c-3p and COL1A2 in ccRCC. “*” represents P-value less than 0.05. Three horizontal lines in the box plot represent minimum, median and maximum, respectively.

Next, we explored the potential functional targets of DUXAP8 and DUXAP9. Genes with higher node degree usually have stronger functions. Therefore, the top 10 hub genes (GAPDH, VEGFA, PTEN, JUN, MMP2, SIRT1, ITGB1, CDC42, COL1A1 and COL1A2) were selected for subsequent analysis. The prognostic values of the top 10 hub genes in human ccRCC were first assessed by Kaplan-Meier plotter. As shown in Table 4, high expression of GAPDH, COL1A1 and COL1A2 indicated a poor prognosis in patients with ccRCC. Four hub genes (PTEN, SIRT1, ITGB1 and CDC42) were favorable prognostic biomarkers in ccRCC. For VEGFA, JUN and MMP2, no prognostic values of them in ccRCC were discovered. Subsequently, we analyzed the expression relationship between DUXAP8/DUXAP9 and the top 10 hub genes (Table 5). The analytic result showed that six hub genes (GAPDH, PTEN, MMP2, ITGB1, COL1A1 and COL1A2) and five hub genes (PTEN, MMP2, ITGB1, COL1A1 and COL1A2) were positively correlated with DUXAP8 and DUXAP9, respectively. By combination of ceRNA hypothesis and the results from survival analysis and correlation analysis, we suggested that COL1A1 and COL1A2 may be two most potential functional targets of DUXAP8 and DUXAP9. Then, we evaluated the expression levels of the two target genes in ccRCC using two online databases, GEPIA and UALCAN. As shown in Figure 6A and Figure 6B, both COL1A1 and COL1A2 were significantly upregulated in cancer samples compared with normal samples. A similar analytic result was acquired when we determined COL1A1 and COL1A2 expression using UALCAN database (Figure 6C and Figure 6D). Furthermore, COL1A1 and COL1A2 expression among various major stages showed statistical difference as presented in Figure 6E and Figure 6F, respectively. Generally, they were markedly upregulated in advanced stages of ccRCC. Finally, the expression correlation of miR-29c-3p with COL1A1/COL1A2 was assessed. Figure 6G and Figure 6H demonstrated that miR-29c-3p was negatively associated with COL1A1 and COL1A2 expression in ccRCC.

**Table 4:** Prognostic values of top ten hub genes in clear cell renal cell carcinoma (ccRCC) determined by Kaplan-Meier plotter.

**Gene names**	**P-value**	**HRs^a^**	**95%CI^b^**	**Prognostic outcomes**
**GAPDH**	**3.00E-04**	**1.77**	**1.29–2.42**	**Poor**
VEGFA	0.16	0.79	0.57**–**1.10	Good
PTEN	9.00E-05	0.56	0.41**–**0.75	Good
JUN	0.0587	0.75	0.55**–**1.01	Good
MMP2	0.28	1.19	0.87**–**1.64	Poor
SIRT1	5.00E-06	0.47	0.34**–**0.65	Good
ITGB1	2.40E-04	0.57	0.43**–**0.78	Good
CDC42	5.60E-08	0.45	0.33**–**0.60	Good
**COL1A1**	**0.00042**	**1.73**	**1.27–2.35**	**Poor**
**COL1A2**	**0.0098**	**1.55**	**1.11-2.17**	**Poor**

^a^HRs: hazard ratios;

^b^95%CI: 95% confidence interval.

**Table 5:** Correlation analysis between DUXAP8/DUXAP9 with top 10 hub genes expression in kidney renal clear cell carcinoma using GEPIA database.

**Gene**	**DUXAP8**		**DUXAP9**	
	**R**	**P-value**	**R**	**P-value**
GAPDH	0.1300	0.0032	0.0970	0.0270
VEGFA	0.0200	0.6500	0.0960	0.0290
**PTEN**	**0.1500**	**0.0006**	**0.1600**	**0.0002**
JUN	−0.0390	0.3700	**–**0.0290	0.5200
**MMP2**	**0.4100**	**0.0000**	**0.3600**	**0.0000**
SIRT1	**–**0.0260	0.5500	0.0079	0.8600
**ITGB1**	**0.2400**	**0.0000**	**0.2500**	**0.0000**
CDC42	0.0360	0.4100	0.0630	0.1500
**COL1A1**	**0.3600**	**0.0000**	**0.3000**	**0.0000**
**COL1A2**	**0.4300**	**0.0000**	**0.3600**	**0.0000**

### DUXAP8/DUXAP9-miR-29c-3p-COL1A1/COL1A2 axis controls growth of renal cell carcinoma

**Figure 7:**
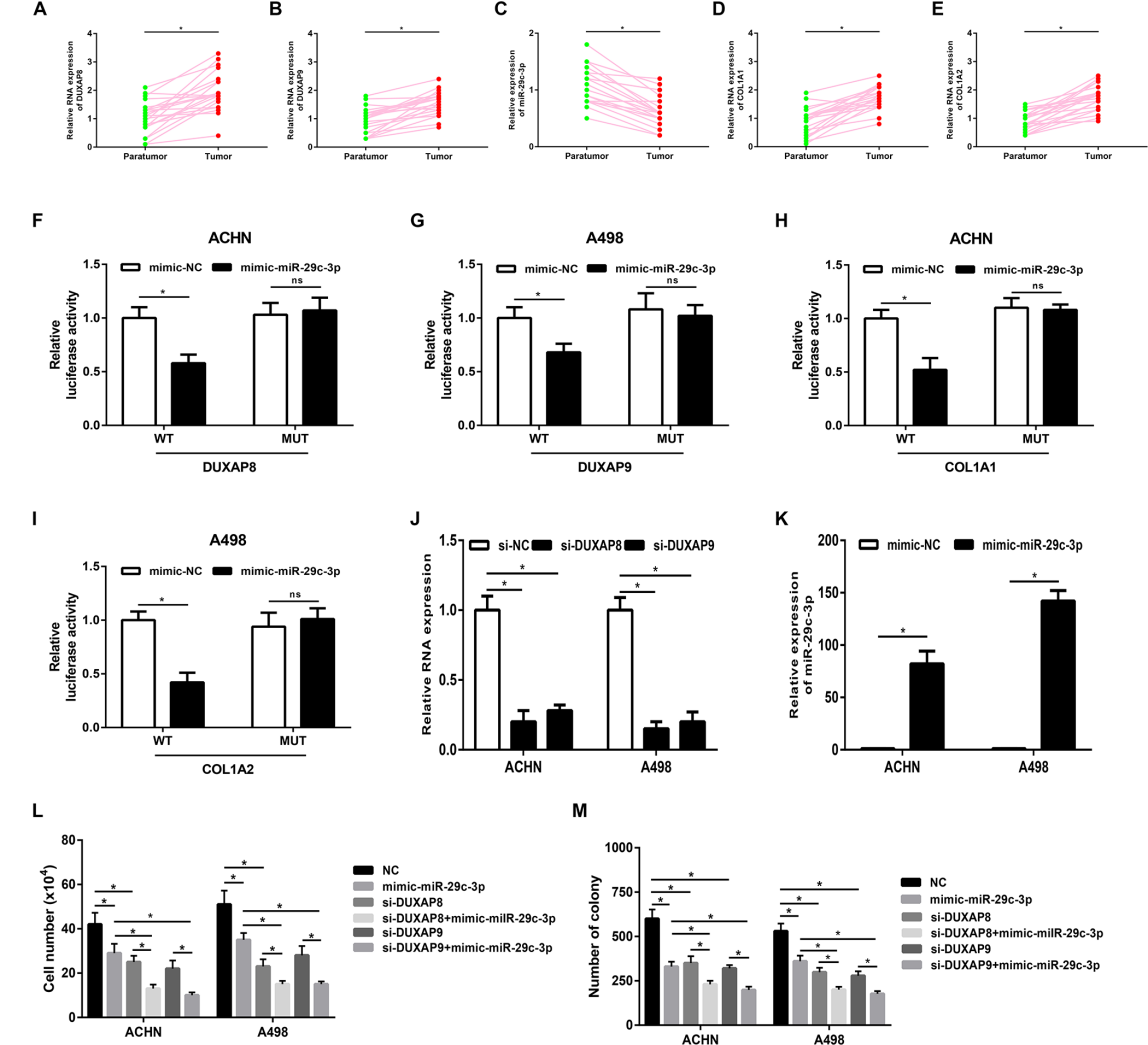
**Pseudogenes DUXAP8 and DUXAP9 promote tumor growth *via* suppression of miR-29c-3p-COL1A1/COL1A2 axis in renal cell carcinoma**. (**A**) The expression of pseudogene DUXAP8 in renal cell carcinoma samples and normal controls; (**B**) the expression of pseudogene DUXAP9 in renal cell carcinoma samples and normal controls; (**C**) the expression of miR-29c-3p in renal cell carcinoma samples and normal controls; (**D**) the expression of COL1A1 in renal cell carcinoma samples and normal controls; (**E**) the expression of COL1A2 in renal cell carcinoma samples and normal controls; (**F**) miR-29c-3p suppressed *Renilla* luciferase activity of the reporters containing the wild-type but not mutant DUXAP8 in ACHN cell line; (**G**) miR-29c-3p suppressed *Renilla* luciferase activity of the reporters containing the wild-type but not mutant DUXAP9 in A498 cell line; (**H**) miR-29c-3p suppressed *Renilla* luciferase activity of the reporters containing the wild-type but not mutant COL1A1 in ACHN cell line; (**I**) miR-29c-3p suppressed *Renilla* luciferase activity of the reporters containing the wild-type but not mutant COL1A2 in A498 cell line; (**J**) the knockdown effect of si-DUXAP8 and si-DUXAP9 in ACHN and A498 cell lines; (**K**) the overexpression effect of miR-29c-3p mimic in ACHN and A498 cell lines; (**L**) knockdown of pseudogene DUXAP8/DUXAP9 or/and overexpression of miR-29c-3p inhibited cell growth *in vitro*; (**M**) knockdown of pseudogene DUXAP8/DUXAP9 or/and overexpression of miR-29c-3p suppressed the colony formation of cell lines in renal cell carcinoma. “*” represents P-value less than 0.05. Abbreviations: MUT, mutant; WT, wild type.

**Figure 8:**
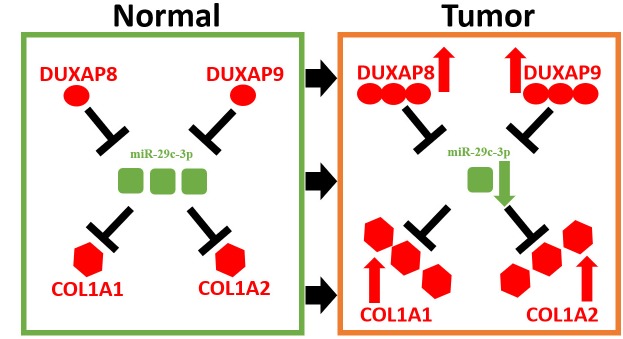
**Model of DUXAP8/DUXAP9-miR-29c-3p-COL1A1/COL1A2 axis in renal cell carcinoma.**

These bioinformatic analytic findings indicate that pseudogene DUXAP8 and DUXAP9 may fuel onset and development of renal cell carcinoma by targeting COL1A1 and COL1A2 through competitively binding miR-29c-3p. In this part, we conducted corresponding experimental validation. Firstly, we determined the expression levels of five constituents (DUXAP8, DUXAP9, miR-29c-3p, COL1A1 and COL1A2) in 20 pairs of clinical cancer tissues and normal tissues of renal cell carcinoma. As shown in Figure 7A, 7B, two pseudogenes DUXAP8 and DUXAP9 were significantly upregulated in cancer samples compared with normal samples. miR-29c-3p expression level (Figure 7C) was markedly lower but COL1A1 (Figure 7D) and COL1A2 (Figure 7E) expression levels were higher in cancer samples than that in normal samples. Next, luciferase reporter assay was introduced to affirm the direct bind of miR-29c-3p to DUXAP8 (Figure 7F), DUXAP9 (Figure 7G), COL1A1 (Figure 7H) and COL1A2 (Figure 7I). Subsequently, we employed siRNAs of DUXAP8/ DUXAP9 to knockdown DUXAP8/DUXAP9 and used mimic of miR-29c-3p to upregulate miR-29c-3p. The knockdown and overexpression effects were presented in Figure 7J and Figure 7K, respectively. Finally, we detected proliferative roles of DUXAP8, DUXAP9 and miR-29c-3p in renal cell carcinoma cell lines. As shown in Figure 7L, knockdown of DUXAP8 or DUXAP9 could significantly suppress cell growth and overexpression of miR-29c-3p also inhibited cell growth. Moreover, we found that cells with knockdown of DUXA8/DUXAP9 and overexpression of miR-29c-3p grew the slowest. Identical with cell counting assay, colony formation assay demonstrated downregulation of DUXAP8/ DUXAP9 or upregulation of miR-29c-3p decreased colony number (Figure 7M). Taken together, amplification of pseudogenes DUXAP8 and DUXAP9 may lead to increase expression of COL1A1 and COL1A2 by competitively binding to miR-29c-3p, resulting in uncontrolled cell proliferation in renal cell carcinoma (Figure 8).

## DISCUSSION

For a long time, pseudogenes are considered as non-functional or ‘junk’ DNA. Growing studies have suggested that pseudogenes act as ceRNAs of functional genes by competitively binding to shared miRNAs, thereby regulating a variety of physiological and pathological processes, including carcinogenesis [23–25]. However, pseudogenes and their underlying ceRNA regulatory mechanisms in RCC remain largely unknown. To the best of our knowledge, a pseudogene-miRNA-target gene network in renal cell carcinoma is still not been constructed.

In this study, we first screened several differentially expressed pseudogenes in ccRCC using the dreamBase database, after which expression levels of these pseudogenes were further validated using another database (GEPIA). Finally, a total of 47 pseudogenes, consisting of 31 upregulated and 16 downregulated pseudogenes in ccRCC were identified. Subsequently, survival analysis and differential expression analysis among various major stages demonstrated that only high expression of pseudogene DUXAP8 and DUXAP9 indicated poor OS and RFS in ccRCC, chRCC and pRCC. DUXAP8 has been reported to function as an oncogene in glioma [27], pancreatic cancer [14], bladder cancer [28, 29], gastric cancer [30], non-small-cell lung cancer [31] as well as renal cell carcinoma [32]. DUXAP9 has also been found to promote non-small-cell lung cancer progression by directly binding with Cbl-b and thus augmenting EGFR signaling [33]. All these reports together with the previous analytic results suggest that pseudogene DUXAP8 and DUXAP9 may serve as two promising therapeutic targets ad prognostic biomarkers for RCC.

ceRNA hypothesis is an important action mechanism of ncRNAs, including pseudogenes [22]. Therefore, we predicted potential miRNAs that can theoretically bind to pseudogene DUXAP8 and DUXAP9 using starBase database. 33 and 5 potential miRNAs were predicted to bind to DUXAP8 and DUXAP9, respectively. By combination of correlation, expression and survival analysis for these miRNAs, miR-29c-3p was identified as the most potential binding miRNA of DUXAP8 and DUXAP9. It has been well documented that miR-29c-3p is an anti-tumor miRNA in multiple human cancers, such as hepatocellular carcinoma [34], breast cancer [35], gastric cancer [36], pancreatic cancer [37] as well as renal cell carcinoma [38]. Next, we predicted the downstream target genes of miR-29c-3p by miRNet, and 254 target genes were obtained. To further understand and explore the roles of DUXAP8 and DUXAP9 in RCC, pathway enrichment analysis for these target genes were performed. The results demonstrated that these target genes were significantly enriched in renal cell carcinoma and some cancer-associated pathways, including extracellular matrix organization.

It has been widely acknowledged that genes with more node degrees usually possess more functions. The top 10 hub genes among the PPI network of target genes of miR-29c-3p were selected for further analysis. Based on ceRNA mechanism, pseudogenes’ expression levels and prognostic values should be positively correlated with their functional targets. After performing survival analysis and correlation analysis, only two hub genes (COL1A1 and COL1A2) were met this requirement. The expression levels of COL1A1 and COL1A2 in ccRCC were obviously higher than that in normal controls, further suggesting that COL1A1 and COL1A2 may be two potential functional targets of DUXAP8 and DUXAP9. Previous studies have reported that COL1A1 and COL1A2 could enhance cancer progression. For example, Liu et al. suggested that COL1A1 promoted metastasis of breast cancer [39]; Zhang et al. found that COL1A1 facilitated metastasis by regulating the WNT/PCP pathway in colorectal cancer [40]; Yu et al. confirmed that COL1A2 inhibited colorectal cancer cell proliferation, migration and invasion [41]. The group of Li J also supposed that COL1A1 and COL1A2 might predict poor clinical outcomes in gastric cancer patients [42]. Moreover, Yamada et al. reported that high expression of ten target genes of miR-29 family, including COL1A1, predicted poor patient prognosis in renal cell carcinoma patients [43]. Subsequently, correlation analysis revealed that miR-29c-3p expression was inversely linked to COL1A1 and COL1A2 expression. All these findings suggest that pseudogene DUXAP8/DUXAP9-miR-29c-3p-COL1A1/COL1A2 may be a critical signaling pathway in onset and progression of renal cell carcinoma. qPCR showed that DUXAP8, DUXAP9, COL1A1 and COL1A2 were significantly upregulated in renal cell carcinoma cancer samples compared with normal controls whereas miR-29c-3p expression in cancer tissues was decreased. miR-29c-3p was confirmed to directly bind to DUXAP8, DUXAP9, COL1A1 and COL1A2 by luciferase reporter assay. Moreover, functional experiments demonstrated that knockdown of pseudogenes DUXAP8/DUXAP9 reduced proliferative ability of renal cell carcinoma. All these findings were in accordance with our previous analytic results, further partially suggesting accuracy of these bioinformatic analyses

Although *in silico* analyses and preliminary experimental validation have shown potential oncogenic roles of DUXAP8 and DUXAP9 and its underlying ceRNA regulatory mechanism in renal cell carcinoma, much more experiments and big clinical trials need to be launched to further confirm these preliminary data in the future.

## MATERIALS AND METHODS

### Screening for differentially expressed pseudogene

RNA-seq expression data of pseudogenes in human ccRCC were directly downloaded from the dreamBase database (http://rna.sysu.edu.cn/dreamBase/index.php). DreamBase database is an integrated platform for analyzing regulatory features of pseudogenes from multi-dimensional high-throughput sequencing data [44]. |log_2_FC| > 2.0 was set as the cut-off criterion for differentially expressed pseudogenes. FC = Tumor/ Normal. Subsequently, they were validated by pseudogene names and expression levels using dreamBase database again. These screened pseudogenes were considered as candidate pseudogenes.

### Confirmation pseudogene expression

A newly developed interactive tool, named Gene Expression Profiling Interactive Analysis (GEPIA) (http://gepia.cancer-pku.cn/detail.php), provides customizable functions such as tumor/normal differential expression analysis, profiling according to cancer types or pathological stages, patient survival analysis, similar gene detection, correlation analysis and dimensionality reduction analysis [45].

In this study, GEPIA was utilized to further determine expression levels of candidate pseudogenes in human KIRC. P-value < 0.05 and |log_2_FC| > 1.0 were set as the thresholds for identifying potential pseudogenes. Expression differences among various major stages of these potential pseudogenes were also studied using this database. The target name was entered in the “stage plot” page, with selection of “Yes” for “use major stage” and “log scale”. After click the “Plot”, the stage plot and corresponding F-value and P-value were automatically generated. P-value < 0.05 was considered as statistically significant.

### Kaplan-Meier survival analysis

The prognostic values (OS, overall survival; RFS, disease free survival) of potential pseudogenes in patients with ccRCC, chRCC and pRCC were assessed *via* GEPIA database (http://gepia.cancer-pku.cn/detail.php) [45]. Longrank P-value < 0.05 was considered as statistically significant. The pseudogenes with significant OS and RFS in ccRCC, chRCC and pRCC were re-defined as key pseudogenes and were chosen for subsequent investigation.

### Prediction of miRNA binding to pseudogene

The potential miRNA-pseudogene interactions were predicted by starBase (version 3) (http://starbase.sysu.edu.cn/), which is an open-source platform for studying the miRNA-ncRNA, miRNA-mRNA, ncRNA-RNA, RNA-RNA, RBP-ncRNA and RBP-mRNA interactions from CLIP-seq, degradome-seq and RNA-RNA interactome data [46, 47]. The predicted miRNAs could be directly exported as excel, after which a visual pseudogene-miRNA network was established using Cytoscape software (version 3.6.0).

### Correlation analysis between pseudogene/gene and miRNA

StarBase (version 3) (http://starbase.sysu.edu.cn/) was also utilized to perform correlation analysis between pseudogene and miRNA in ccRCC [46, 47]. The pseudogene-miRNA interactions with R < -0.1 AND P-value < 0.05 was considered as potential items. Accordingly, these miRNAs involved in the potential interactions were defined as potential miRNAs. This database was also employed to analyze the expression association between miRNA and gene. Similarly, miRNA-gene interactions with R < -0.1 and P-value < 0.05 were regarded as significant.

### Expression analysis of potential miRNA

The expression levels of potential miRNAs in ccRCC, chRCC and pRCC were also analyzed by starBase (version 3) (http://starbase.sysu.edu.cn/) [46, 47]. P < 0.05 was considered as statistically significant.

### Survival analysis of potential miRNA

The prognostic values of miRNAs in ccRCC and pRCC were determined through Kaplan-Meier plotter database (http://kmplot.com/analysis/), which is capable to access the effect of 11,000 miRNAs from 20 different cancer types, including clear cell carcinoma [48, 49]. Simply, the miRNAs were first typed into this database. Then, Kaplan Meier survival plots were directly created, and logrank P-value, hazard ratio (HR) and 95% confidence interval (CI) were displayed on the webpage. Logrank P-value < 0.05 was considered as statistically significant.

### Prediction of potential target gene of miRNA

miRNet is an easy-to-use tool with comprehensive support for statistical analysis and functional interpretation of data generated in miRNAs studies. In this database, the miRNA target gene data were collected from several well-annotated databases, including miRTarBase v7.0, TarBase v7.0 and miRecords [50, 51]. Therefore, miRNet was selected to predict potential downstream target genes of potential miRNAs.

### Gene Ontology (GO) and Kyoto Encyclopedia of Genes and Genomes (KEGG) pathway analysis

Enrichment analysis for predicted target genes was conducted using Enrichr database (http://amp.pharm.mssm.edu/Enrichr/), which is a comprehensive gene set enrichment analysis web server [52, 53]. KEGG pathway analysis and three categories of GO functional annotation, including biological process (BP), cellular component (CC) and molecular function (MF), were included in this study. The bar charts of BP, CC, MF and KEGG were directly downloaded from the webpage.

### Establishment and analysis of protein-protein interaction (PPI) network

STRING database (http://string-db.org) is a database of known and predicted PPIs, in which interactions are derived from five main sources: genomic context predictions, high-throughput lab experiments, (conserved) co-expression, automated text mining and previous knowledge in databases [54, 55]. PPI network of predicted target genes was constructed using STRING database. Firstly, these predicted targets were mapped into this database. Then, gene pairs with combined score >= 0.4 were exported. Subsequently, these gene pairs were re-loaded into Cytoscape software (version 3.6.0), after which the hub genes among the PPI network were identified using Cytohubba plugin of Cytoscape software (version 3.6.0) as previously described [56].

### Correlation analysis between pseudogene and hub gene

To determine the most potential target of pseudogene, the correlation analysis between pseudogene and hub gene in ccRCC was performed *via* GEPIA database (http://gepia.cancer-pku.cn/detail.php) [45]. Pseudogene -hub gene pairs with R > 0.1 and P-value < 0.05 were considered as potential pairs and were selected for further analysis.

### Survival analysis of hub gene

The prognostic values of hub genes in ccRCC were determined using Kaplan-Meier plotter database (http://kmplot.com/analysis/) as mentioned above. Logrank P-value < 0.05 was considered as statistically significant.

### Expression analysis of hub gene

Two online databases, named GEPIA and UALCAN, were introduced to detect the expression levels of hub genes in ccRCC [45, 57]. The expression differences among different major stages of hub genes in clear cell carcinoma were also evaluated by GEPIA database. P-value < 0.05 was regarded as statistically significant.

### Cell culture and clinical samples

The human renal cell carcinoma cell lines ACHN and A498 were purchased from ATCC. The cells were maintained in minimal essential medium (MEM) supplemented with 10% FBS, penicillin (100 U/ml) and streptomycin (100ug/ml). 20 pairs of clear cell renal cell carcinoma tumor tissues and adjacent normal kidney tissues were collected from the First Affiliated Hospital of Zhejiang University. All procedures performed in this study involving human participants were conducted in accordance with the ethical standards of the First Affiliated Hospital of Zhejiang University and written informed consent from every participant was obtained.

### Primer, RNA oligoribonucleotide, miRNA mimic and vectors

ALL primers, RNA oligoribonucleotides and miRNA mimic used in this study were designed and purchased from RiboBio Co. Ltd (Guangzhou, China).

### Analysis of gene or miRNA expression

The expression level of DUXAP8, DUXAP9, miR- 29c-3p, COL1A1 and COL1A2 was analyzed by quantitative real-time PCR as we previously described [58].

### Cell transfection

RNA oligios and miRNA mimic were transfected into cells with a final concentration of 50 nM using Lipofectamine^TM^ 3000 (Invitrogen) according to manufacturer’s instruction.

### Luciferase reporter assay

To determine if miIR-29c-3p can directly bind to DUXAP8, DUXAP9, COL1A1 and COL1A2, luciferase reporter assay was performed as we described previously [59].

### Cell counting assay

Cell counting assay was employed to evaluate cell growth. Cells were first transfected as mentioned above. At 24 h post-transfection, 4 x 10^4^ cells were re-seeded into 12-well plates. After culturing for 3 days, cell numbers were counted.

### Colony formation assay

Twenty-four hours after transfection, 500 of transfected cells (ACHN and A498) were re-seeded into 6 well-plates and cultured for two weeks. Two weeks later, colonies were fixed in methanol for 30 minutes and then stained with a 0.1% crystal violet solution for 15 minutes. Finally, visible colonies were counted.

### Statistical analysis

Most of the statistical analysis has been done by the bioinformatic tools mentioned above. Experimental results were shown as mean±SD. Paired Student’s *t*-test was used to evaluate expression differences of genes or miRNA between paratumor group and tumor group. Differences between two groups were estimated by unpaired Student’s *t*-test. A two-tailed value of P < 0.05 was considered as statistically significant.

## CONCLUSIONS

To sum up, this study for the first time comprehensively and systematically explored the function and ceRNA regulatory mechanism of pseudogenes in renal cell carcinoma. Our current results indicated that DUXAP8 and DUXAP9 may act as two key oncogenes in renal cell carcinoma through upregulation of COL1A1 and COL1A2 by competitively binding miR-29c-3p. Moreover, our results suggested that DUXAP8 and DUXAP9 and other components in this pathway possessed significant prognostic values in patients with kidney cancer. All these findings are worthy of more experimental validation in the future.

## Supplementary Material

Supplementary Figure

Supplementary Tables

Supplementary Table 1

Supplementary Table 4
