# Supplementary material for: Overexpressed pseudogenes, DUXAP8 and DUXAP9, promote growth of renal cell carcinoma and serve as unfavorable prognostic biomarkers
Source: Aging (Albany NY). 2019 Aug 13;11(15):5666–88. doi: 10.18632/aging.102152 (PMC6710046; doi:10.18632/aging.102152)
Supplement: Supplementary Figure [file aging-11-102152-s004.pdf]

SUPPLEMENTARY FIGURE

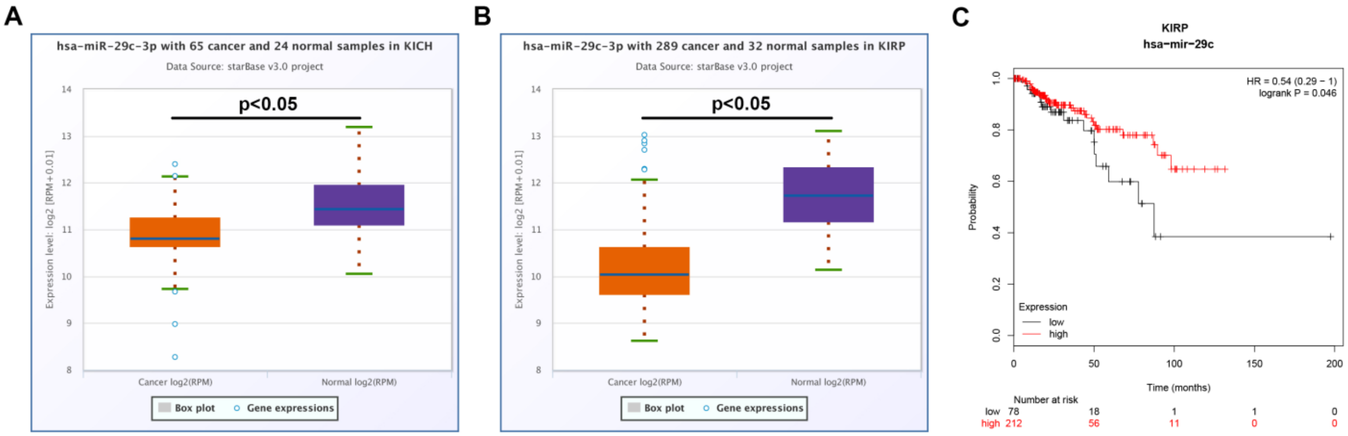

Supplementary Figure 1. Expression and prognostic values of hsa-miR-29c-3p in chRCC (KICH) and pRCC (KIRP). (**A**) Expression level of hsa-miR-29c-3p in chRCC; (**B**) expression level of hsa-miR-29c-3p in pRCC; (**C**) prognostic value of hsa-miR-29c-3p in pRCC. "p<0.05" represents statistically significant.
