# Supplementary material for: Overexpressed pseudogenes, DUXAP8 and DUXAP9, promote growth of renal cell carcinoma and serve as unfavorable prognostic biomarkers
Source: Aging (Albany NY). 2019 Aug 13;11(15):5666–88. doi: 10.18632/aging.102152 (PMC6710046; doi:10.18632/aging.102152)
Supplement: Supplementary Tables [file aging-11-102152-s003.pdf]

SUPPLEMENTARY TABLES

Please browse Full Text version to see the data of Supplementary Tables 1 and 4:

Supplementary Table 1. Dysregulated pseudogenes in clear cell renal cell carcinoma (ccRCC) from dreamBase database.

Supplementary Table 2. Dysregulated pseudogenes in clear cell renal cell carcinoma (ccRCC) identified by GEPIA database.

Upregulated pseudogenes	Downregulated pseudogenes
RP5-1120P11.4	RP11-480I12.5
AC007326.9	NUDT4P2
RP4-631H13.6	ZNF300P1
APOC1P1	RP11-255H23.2
RP11-155G14.5	AF186192.5
DGCR5	TUBBP5
FER1L4	RP11-231P20.2
RP11-255M6.1	FOLH1B
HSPA7	COX7A2P1
KRT223P	PPP1R36
FCGR2C	TPTEP1
PTP4A2P2	MST1L
HLA-J	AP000344.4
HLA-DPB2	LINC00982
OR2I1P	SLC2A3P1
LILRA6	RP11-12A20.10
CYP21A1P	
RP11-693N9.2	
DUXAP8	
SDHAP3	
NCF1B	
NCF1C	
OR7E47P	
DUXAP9	
AC093616.4	
RP11-75L1.2	
RP11-64B16.2	
GGTA1P	
NBPF8	
NAPSB	

Supplementary Table 3. The prognostic values of dysregulated pseudogenes in kidney clear cell carcinoma determined by GEPIA database.

Upregulated pseudogene	OS[a]	RFS[b]	Downregulated pseudogene	OS	RFS
RP5-1120P11.4	No	Good	RP11-480I12.5	Good	No
**AC007326.9**	**Poor**	**Poor**	**NUDT4P2**	**Good**	**Good**
RP4-631H13.6	No	No	ZNF300P1	No	No
APOC1P1	Poor	No	**RP11-255H23.2**	**Good**	**Good**
RP11-155G14.5	No	No	**AF186192.5**	**Good**	**Good**
DGCR5	No	No	TUBBP5	No	Good
FER1L4	Poor	No	RP11-231P20.2	Good	No
RP11-255M6.1	No	No	FOLH1B	Good	No
HSPA7	Poor	No	COX7A2P1	No	No
KRT223P	No	Poor	PPP1R36	No	No
FCGR2C	No	No	TPTEP1	No	No
PTP4A2P2	Good	Good	MST1L	No	Good
HLA-J	No	No	AP000344.4	No	No
HLA-DPB2	No	No	LINC00982	No	Good
OR2I1P	No	No	**SLC2A3P1**	**Good**	**Good**
LILRA6	No	No	RP11-12A20.10	No	No
CYP21A1P	Poor	No			
RP11-693N9.2	No	No			
**DUXAP8**	**Poor**	**Poor**			
SDHAP3	No	No			
NCF1B	No	No			
NCF1C	No	No			
OR7E47P	Good	Good			
**DUXAP9**	**Poor**	**Poor**			
AC093616.4	No	No			
RP11-75L1.2	Poor	No			
RP11-64B16.2	No	No			
GGTA1P	No	No			
NBPF8	Poor	No			
NAPSB	No	No			

[a]OS, overall survival;
[b]RFS, disease free survival.

Supplementary Table 4. Potential target genes of has-miR-29c-3p predicted by miRNet database.
