# Supplementary material for: Overexpressed pseudogenes, DUXAP8 and DUXAP9, promote growth of renal cell carcinoma and serve as unfavorable prognostic biomarkers
Source: Aging (Albany NY). 2019 Aug 13;11(15):5666–88. doi: 10.18632/aging.102152 (PMC6710046; doi:10.18632/aging.102152)
Supplement: Supplementary Table 1 [file aging-11-102152-s002.docx]

**Supplementary Tables 1.** Dysregulated pseudogenes in clear cell renal cell carcinoma (ccRCC) from dreamBase database.

| Upregulated  pseudogene | Log_2_FC^a^ | Downregulated  pseudogene | Log_2_FC^a^ |
| --- | --- | --- | --- |
| TMSB4XP6 | 8.68 | CETN4P | -2 |
| RP5-1120P11.4 | 7.34 | TMED10P2 | -2.01 |
| AC007326.9 | 6.82 | RP11-740C1.2 | -2.03 |
| RP4-631H13.6 | 6.74 | RPL7AP28 | -2.08 |
| APOC1P1 | 6.44 | RP11-137N23.1 | -2.09 |
| RP11-28B23.1 | 6.21 | RP11-480I12.5 | -2.09 |
| HMGN2P47 | 6.19 | NUDT4P2 | -2.1 |
| STK19B | 6.02 | VN1R51P | -2.18 |
| ZNF37CP | 5.95 | PDZK1P1 | -2.18 |
| HCG4P11 | 5.76 | CELP | -2.18 |
| RP11-242D8.3 | 5.55 | ANOS2P | -2.22 |
| RP11-155G14.5 | 5.51 | AC011330.5 | -2.23 |
| DGCR5 | 5.44 | RP11-720N19.1 | -2.25 |
| RP4-560B9.4 | 5.43 | RP11-1E11.1 | -2.25 |
| AC015977.5 | 5.36 | PRAMENP | -2.25 |
| FER1L4 | 5.31 | BNIP3P4 | -2.25 |
| CTA-313A17.2 | 5.17 | ZNF300P1 | -2.26 |
| HIST1H1PS1 | 5.04 | RP11-480I12.9 | -2.28 |
| CTC-458A3.1 | 5 | RP11-486A14.1 | -2.29 |
| RPS27P29 | 4.91 | RP11-44F14.1 | -2.32 |
| SNRPGP4 | 4.76 | RP11-255H23.2 | -2.34 |
| RP4-742C19.12 | 4.7 | AF186192.5 | -2.34 |
| DEFB109P3 | 4.64 | PDCD6IPP1 | -2.37 |
| AC002075.4 | 4.59 | RP11-574E24.3 | -2.38 |
| BRD9P2 | 4.59 | PABPC1P4 | -2.39 |
| RP11-603B24.1 | 4.59 | RP11-15E1.6 | -2.46 |
| RP11-481A20.10 | 4.52 | LRRC37A6P | -2.49 |
| RP11-25I15.1 | 4.39 | CHMP1B2P | -2.5 |
| AC073850.6 | 4.39 | AC021016.7 | -2.5 |
| GGT8P | 4.39 | FAM60BP | -2.5 |
| RP11-20O24.1 | 4.17 | PTPN20CP | -2.5 |
| TRAV35 | 4.17 | PRSS44 | -2.56 |
| RPS26P47 | 4.17 | GLDCP1 | -2.56 |
| IGHV1OR15-3 | 4.17 | RP11-108M9.3 | -2.56 |
| MLLT10P1 | 4.09 | OR10AB1P | -2.58 |
| AKR7A2P1 | 4 | WTAPP1 | -2.61 |
| AC005363.9 | 4 | TUBBP5 | -2.62 |
| RP11-47G11.2 | 4 | AC112198.1 | -2.68 |
| RP11-255M6.1 | 3.99 | PPP1R14BP2 | -2.71 |
| CTD-3030D20.1 | 3.91 | RP11-231P20.2 | -2.72 |
| IFITM4P | 3.91 | FOLH1B | -2.75 |
| SLC47A1P1 | 3.91 | TAS2R2P | -2.77 |
| UBA52P6 | 3.91 | ADORA2BP1 | -2.85 |
| DUSP5P1 | 3.86 | IFNWP19 | -2.91 |
| CYCSP24 | 3.81 | COX7A2P1 | -2.93 |
| GTF2IP9 | 3.81 | RP11-545A16.3 | -2.94 |
| RPL7P50 | 3.81 | PPP1R36 | -2.97 |
| HMGN2P40 | 3.81 | CTSLP2 | -3.04 |
| HSPA7 | 3.73 | TPTEP1 | -3.18 |
| AC104843.3 | 3.7 | RPSAP52 | -3.18 |
| LGALS17A | 3.7 | AC004870.5 | -3.18 |
| AL590762.11 | 3.7 | RPLP0P2 | -3.19 |
| TIMM8AP1 | 3.7 | AZGP1P1 | -3.23 |
| RPL23AP21 | 3.7 | MST1L | -3.51 |
| RP4-733B9.1 | 3.7 | TUBB8P7 | -3.68 |
| CTD-2528L19.3 | 3.7 | RP11-566K19.5 | -3.79 |
| RP4-539M6.22 | 3.7 | AP000344.4 | -3.84 |
| RP11-587D21.1 | 3.7 | BX842568.2 | -4.32 |
| RPL22P19 | 3.7 | RPL12P30 | -4.55 |
| TTC4P1 | 3.7 | RP11-314N2.2 | -4.56 |
| RP11-397P13.7 | 3.7 | VN1R85P | -4.86 |
| AC011933.2 | 3.59 | LINC00982 | -5.83 |
| RPL35AP32 | 3.59 | SLC2A3P1 | -5.87 |
| RP11-146E13.5 | 3.59 | RP11-12A20.10 | -6.35 |
| PTMAP1 | 3.46 |  |  |
| TXNP6 | 3.46 |  |  |
| RPS14P8 | 3.46 |  |  |
| HIST1H2BO | 3.46 |  |  |
| NIPA2P1 | 3.46 |  |  |
| EIF5AP3 | 3.46 |  |  |
| RP11-247I13.3 | 3.46 |  |  |
| FTH1P22 | 3.43 |  |  |
| CTD-2114J12.1 | 3.39 |  |  |
| KRT223P | 3.38 |  |  |
| FCGR2C | 3.36 |  |  |
| UBTFL6 | 3.35 |  |  |
| ADGRE4P | 3.32 |  |  |
| RP11-347C12.12 | 3.32 |  |  |
| RPS15AP16 | 3.32 |  |  |
| RP1-13D10.2 | 3.32 |  |  |
| RPL7AP7 | 3.32 |  |  |
| MRPS18AP1 | 3.32 |  |  |
| RP11-575G13.2 | 3.32 |  |  |
| VN1R42P | 3.32 |  |  |
| CPHL1P | 3.32 |  |  |
| RPL31P49 | 3.32 |  |  |
| RPS26P28 | 3.32 |  |  |
| SCOCP1 | 3.32 |  |  |
| HMGB3P32 | 3.32 |  |  |
| RP11-493L12.6 | 3.32 |  |  |
| AC006946.12 | 3.32 |  |  |
| TRBV21-1 | 3.32 |  |  |
| FTLP15 | 3.32 |  |  |
| RPS26P11 | 3.32 |  |  |
| CXCR2P1 | 3.3 |  |  |
| NKAIN1 | 3.26 |  |  |
| RPS20P4 | 3.17 |  |  |
| ITM2BP1 | 3.17 |  |  |
| CTD-2651B20.4 | 3.17 |  |  |
| RP11-613E4.5 | 3.17 |  |  |
| HNRNPA1P21 | 3.17 |  |  |
| CTD-2262B20.1 | 3.17 |  |  |
| BTG1P1 | 3.17 |  |  |
| RPL18P10 | 3.17 |  |  |
| RP1-89D4.1 | 3.17 |  |  |
| PMS2P9 | 3.17 |  |  |
| BNIP3P22 | 3.17 |  |  |
| OACYLP | 3.08 |  |  |
| RP11-496I2.5 | 3.06 |  |  |
| RPL7L1P9 | 3 |  |  |
| RAP1AP | 3 |  |  |
| AC012066.1 | 3 |  |  |
| RP1-164F3.8 | 3 |  |  |
| RPL29P24 | 3 |  |  |
| RP11-250H24.2 | 3 |  |  |
| RP1-13D10.3 | 3 |  |  |
| TNXA | 3 |  |  |
| NUTF2P6 | 3 |  |  |
| RPL31P61 | 3 |  |  |
| RPL21P13 | 3 |  |  |
| RP11-732A19.1 | 3 |  |  |
| CTD-2062A1.2 | 3 |  |  |
| RP11-707G14.8 | 3 |  |  |
| RP11-468N14.09 | 3 |  |  |
| RP11-382A20.1 | 3 |  |  |
| HCG4P8 | 3 |  |  |
| HMGB3P4 | 3 |  |  |
| RSL24D1P11 | 3 |  |  |
| PTP4A2P2 | 2.97 |  |  |
| AC108463.2 | 2.96 |  |  |
| HLA-J | 2.94 |  |  |
| HLA-DPB2 | 2.94 |  |  |
| OR2I1P | 2.9 |  |  |
| RP11-401N16.2 | 2.81 |  |  |
| RPSAP26 | 2.81 |  |  |
| RP4-612B18.3 | 2.81 |  |  |
| BNIP3P27 | 2.81 |  |  |
| BNIP3P26 | 2.81 |  |  |
| RP11-353N4.5 | 2.81 |  |  |
| RP4-641G12.3 | 2.81 |  |  |
| CH17-78J1.1 | 2.81 |  |  |
| EXOSC3P1 | 2.81 |  |  |
| NBEAP3 | 2.81 |  |  |
| CARD17 | 2.81 |  |  |
| RP11-1094M14.8 | 2.81 |  |  |
| AC011515.2 | 2.81 |  |  |
| TPT1P5 | 2.81 |  |  |
| RP11-565J7.1 | 2.81 |  |  |
| RP11-848G14.5 | 2.79 |  |  |
| DLGAP5 | 2.79 |  |  |
| LILRA6 | 2.77 |  |  |
| CYP21A1P | 2.75 |  |  |
| CTSL3P | 2.7 |  |  |
| RP11-578F21.2 | 2.59 |  |  |
| RPL4P1 | 2.59 |  |  |
| RP11-260N14.1 | 2.59 |  |  |
| LL0XNC01-116E7.1 | 2.59 |  |  |
| AC090804.1 | 2.59 |  |  |
| RP11-404O13.4 | 2.59 |  |  |
| RP4-673D20.3 | 2.59 |  |  |
| CBX3P7 | 2.59 |  |  |
| IMMP1LP1 | 2.59 |  |  |
| GS1-304P7.1 | 2.59 |  |  |
| UGT1A2P | 2.59 |  |  |
| BNIP3P10 | 2.59 |  |  |
| RP11-757O6.6 | 2.59 |  |  |
| RPL21P32 | 2.59 |  |  |
| NDUFB8P2 | 2.59 |  |  |
| RP11-644F5.15 | 2.59 |  |  |
| RP11-142C4.4 | 2.59 |  |  |
| RPL23AP43 | 2.59 |  |  |
| RPL21P116 | 2.59 |  |  |
| BMS1P22 | 2.59 |  |  |
| AC068279.3 | 2.59 |  |  |
| PRMT1P1 | 2.59 |  |  |
| STMN1P1 | 2.59 |  |  |
| RP11-79L9.2 | 2.59 |  |  |
| CTD-2301A4.3 | 2.59 |  |  |
| GXYLT1P5 | 2.59 |  |  |
| RP11-382J24.2 | 2.59 |  |  |
| NT5CP1 | 2.59 |  |  |
| RP11-348H3.2 | 2.59 |  |  |
| bP-21264C1.1 | 2.57 |  |  |
| RP11-693N9.2 | 2.54 |  |  |
| DUXAP8 | 2.54 |  |  |
| SNX18P12 | 2.49 |  |  |
| LA16c-60H5.7 | 2.48 |  |  |
| RP11-424C20.2 | 2.46 |  |  |
| RP11-686D22.10 | 2.46 |  |  |
| SDHAP3 | 2.45 |  |  |
| NCF1B | 2.45 |  |  |
| POU5F1P3 | 2.42 |  |  |
| CYP2D7 | 2.42 |  |  |
| PFN1P6 | 2.4 |  |  |
| RP11-23J18.1 | 2.4 |  |  |
| NCF1C | 2.4 |  |  |
| UBTFL5 | 2.38 |  |  |
| RP11-302B13.1 | 2.34 |  |  |
| MED28P7 | 2.32 |  |  |
| GPX1P2 | 2.32 |  |  |
| RPS10P3 | 2.32 |  |  |
| CDC42P3 | 2.32 |  |  |
| RP11-89B16.2 | 2.32 |  |  |
| RP11-215D10.1 | 2.32 |  |  |
| CTD-2267D19.4 | 2.32 |  |  |
| RPL9P2 | 2.32 |  |  |
| SPDYE21P | 2.32 |  |  |
| PRADC1P1 | 2.32 |  |  |
| RP11-172F4.2 | 2.32 |  |  |
| RP5-867C24.1 | 2.32 |  |  |
| CTD-2651B20.5 | 2.32 |  |  |
| CTD-2325A15.3 | 2.32 |  |  |
| KIR2DS4 | 2.32 |  |  |
| RPL23AP3 | 2.32 |  |  |
| PRDX2P3 | 2.32 |  |  |
| RPS9P1 | 2.32 |  |  |
| OR7E47P | 2.32 |  |  |
| AK3P5 | 2.32 |  |  |
| YES1P1 | 2.32 |  |  |
| MRPL40P1 | 2.32 |  |  |
| CDC42P5 | 2.32 |  |  |
| RPL12P42 | 2.32 |  |  |
| CTD-2104P17.1 | 2.32 |  |  |
| FCF1P7 | 2.32 |  |  |
| AC010150.1 | 2.32 |  |  |
| HMGB1P11 | 2.32 |  |  |
| RP11-85G20.1 | 2.32 |  |  |
| AC073150.6 | 2.32 |  |  |
| LINC00264 | 2.32 |  |  |
| RPS4XP1 | 2.32 |  |  |
| PPIHP1 | 2.32 |  |  |
| OR11H7 | 2.32 |  |  |
| RPL21P10 | 2.32 |  |  |
| RP11-561N12.5 | 2.32 |  |  |
| BNIP3P16 | 2.32 |  |  |
| RP11-1G11.2 | 2.32 |  |  |
| RP11-1029M24.1 | 2.32 |  |  |
| RP11-452D12.1 | 2.32 |  |  |
| AC090286.2 | 2.32 |  |  |
| SETP1 | 2.32 |  |  |
| FDPSP7 | 2.32 |  |  |
| OR5BA1P | 2.32 |  |  |
| PRDX1P1 | 2.32 |  |  |
| VN1R12P | 2.32 |  |  |
| RPL10P1 | 2.32 |  |  |
| AL590762.7 | 2.32 |  |  |
| SH3GL1P1 | 2.3 |  |  |
| RP13-644M16.1 | 2.28 |  |  |
| HLA-V | 2.26 |  |  |
| DUXAP9 | 2.25 |  |  |
| RP11-974F13.5 | 2.23 |  |  |
| AC093616.4 | 2.23 |  |  |
| RP11-75L1.2 | 2.21 |  |  |
| DDX12P | 2.21 |  |  |
| RP11-368J21.3 | 2.2 |  |  |
| RP11-381E24.1 | 2.19 |  |  |
| ANKRD20A7P | 2.17 |  |  |
| RP11-64B16.2 | 2.17 |  |  |
| GGTA1P | 2.15 |  |  |
| RP11-835E18.4 | 2.13 |  |  |
| AC063976.7 | 2.12 |  |  |
| VWFP1 | 2.12 |  |  |
| CTD-2521M24.4 | 2.09 |  |  |
| ORAOV1P1 | 2.08 |  |  |
| SIGLEC17P | 2.07 |  |  |
| RPL7AP64 | 2.07 |  |  |
| AC024937.6 | 2.06 |  |  |
| LL0XNC01-240C2.1 | 2.06 |  |  |
| NBPF8 | 2.04 |  |  |
| RP13-644M16.5 | 2.02 |  |  |
| RP3-415N12.1 | 2 |  |  |
| RPS3P2 | 2 |  |  |
| FOSL1P1 | 2 |  |  |
| RP4-800G7.3 | 2 |  |  |
| NSFP1 | 2 |  |  |
| RP1-315G1.1 | 2 |  |  |
| OR7E2P | 2 |  |  |
| RP11-2H8.4 | 2 |  |  |
| ANKRD18DP | 2 |  |  |
| CR848007.2 | 2 |  |  |
| PHBP13 | 2 |  |  |
| RPL7P26 | 2 |  |  |
| RP11-311D14.1 | 2 |  |  |
| RP4-718P11.1 | 2 |  |  |
| CENPUP2 | 2 |  |  |
| RP11-434D2.9 | 2 |  |  |
| AC005682.6 | 2 |  |  |
| RPL7AP34 | 2 |  |  |
| LINC01529 | 2 |  |  |
| RAD1P2 | 2 |  |  |
| RP11-632K20.2 | 2 |  |  |
| BTBD10P2 | 2 |  |  |
| MTND4P26 | 2 |  |  |
| PHBP15 | 2 |  |  |
| GOLGA8VP | 2 |  |  |
| ZMYND19P1 | 2 |  |  |
| RPL9P3 | 2 |  |  |
| RP11-571F15.3 | 2 |  |  |
| SMARCE1P6 | 2 |  |  |
| RPL7P18 | 2 |  |  |
| RP11-183G22.1 | 2 |  |  |
| SNX18P26 | 2 |  |  |
| AP000654.5 | 2 |  |  |
| CTC-484M2.1 | 2 |  |  |
| AC018463.4 | 2 |  |  |
| HMGB1P24 | 2 |  |  |
| CTD-2553L13.7 | 2 |  |  |
| POU5F1P4 | 2 |  |  |
| GAPDHP70 | 2 |  |  |
| RPSAP20 | 2 |  |  |
| MTND4P9 | 2 |  |  |
| NAPSB | 2 |  |  |
| RPL7P17 | 2 |  |  |
| PSMD8P1 | 2 |  |  |
| ARGFXP2 | 2 |  |  |
| CTB-78O21.1 | 2 |  |  |
| GLYATL1P1 | 2 |  |  |
| KRT17P8 | 2 |  |  |
| RPS4XP11 | 2 |  |  |
| RP11-630I5.1 | 2 |  |  |
| TREML3P | 2 |  |  |
| AC010642.2 | 2 |  |  |
| FAM172BP | 2 |  |  |
| MICE | 2 |  |  |
| HSPD1P6 | 2 |  |  |
| RP11-568G11.4 | 2 |  |  |
| PGAM1P7 | 2 |  |  |
| KRR1P1 | 2 |  |  |
| CTC-250P20.2 | 2 |  |  |
| AC026150.6 | 2 |  |  |
| AC010091.1 | 2 |  |  |
| RP11-346E8.1 | 2 |  |  |
| YBX1P4 | 2 |  |  |
| AC064836.3 | 2 |  |  |
| RP11-214J9.1 | 2 |  |  |

^a^FC=Fold change=Tumor expression/normal expression
