# Supplementary material for: Overexpressed pseudogenes, DUXAP8 and DUXAP9, promote growth of renal cell carcinoma and serve as unfavorable prognostic biomarkers
Source: Aging (Albany NY). 2019 Aug 13;11(15):5666–88. doi: 10.18632/aging.102152 (PMC6710046; doi:10.18632/aging.102152)
Supplement: Supplementary Table 4 [file aging-11-102152-s001.docx]

**Supplementary Tables 4.** Potential target genes of has-miR-29c-3p predicted by miRNet database.

| ID | Gene | Entrez |
| --- | --- | --- |
| hsa-mir-29c-3p | CRYBG1 | 202 |
| hsa-mir-29c-3p | AKT2 | 208 |
| hsa-mir-29c-3p | AMFR | 267 |
| hsa-mir-29c-3p | BCKDHA | 593 |
| hsa-mir-29c-3p | BCL2 | 596 |
| hsa-mir-29c-3p | CALM3 | 808 |
| hsa-mir-29c-3p | CASP8 | 841 |
| hsa-mir-29c-3p | SERPINH1 | 871 |
| hsa-mir-29c-3p | CCNA2 | 890 |
| hsa-mir-29c-3p | CCND2 | 894 |
| hsa-mir-29c-3p | CCNT2 | 905 |
| hsa-mir-29c-3p | ENTPD1 | 953 |
| hsa-mir-29c-3p | CDC42 | 998 |
| hsa-mir-29c-3p | CDK6 | 1021 |
| hsa-mir-29c-3p | COL1A1 | 1277 |
| hsa-mir-29c-3p | COL1A2 | 1278 |
| hsa-mir-29c-3p | COL3A1 | 1281 |
| hsa-mir-29c-3p | COL4A1 | 1282 |
| hsa-mir-29c-3p | COL4A2 | 1284 |
| hsa-mir-29c-3p | COL5A2 | 1290 |
| hsa-mir-29c-3p | COL6A2 | 1292 |
| hsa-mir-29c-3p | COL7A1 | 1294 |
| hsa-mir-29c-3p | COL10A1 | 1300 |
| hsa-mir-29c-3p | COL15A1 | 1306 |
| hsa-mir-29c-3p | CTNND1 | 1500 |
| hsa-mir-29c-3p | DDX6 | 1656 |
| hsa-mir-29c-3p | DNMT3A | 1788 |
| hsa-mir-29c-3p | DNMT3B | 1789 |
| hsa-mir-29c-3p | DSC2 | 1824 |
| hsa-mir-29c-3p | DUSP2 | 1844 |
| hsa-mir-29c-3p | EMP1 | 2012 |
| hsa-mir-29c-3p | EPHX2 | 2053 |
| hsa-mir-29c-3p | EREG | 2069 |
| hsa-mir-29c-3p | FBN1 | 2200 |
| hsa-mir-29c-3p | FGA | 2243 |
| hsa-mir-29c-3p | FGB | 2244 |
| hsa-mir-29c-3p | FGG | 2266 |
| hsa-mir-29c-3p | FOS | 2353 |
| hsa-mir-29c-3p | FRK | 2444 |
| hsa-mir-29c-3p | GAPDH | 2597 |
| hsa-mir-29c-3p | HDGF | 3068 |
| hsa-mir-29c-3p | HMGCR | 3156 |
| hsa-mir-29c-3p | SLC29A2 | 3177 |
| hsa-mir-29c-3p | ID3 | 3399 |
| hsa-mir-29c-3p | IFRD1 | 3475 |
| hsa-mir-29c-3p | IGFBP1 | 3484 |
| hsa-mir-29c-3p | INSIG1 | 3638 |
| hsa-mir-29c-3p | ITGA6 | 3655 |
| hsa-mir-29c-3p | ITGB1 | 3688 |
| hsa-mir-29c-3p | JUN | 3725 |
| hsa-mir-29c-3p | LAMC1 | 3915 |
| hsa-mir-29c-3p | LAMC2 | 3918 |
| hsa-mir-29c-3p | LIMS1 | 3987 |
| hsa-mir-29c-3p | LOX | 4015 |
| hsa-mir-29c-3p | LRP6 | 4040 |
| hsa-mir-29c-3p | MXD1 | 4084 |
| hsa-mir-29c-3p | MAZ | 4150 |
| hsa-mir-29c-3p | MCL1 | 4170 |
| hsa-mir-29c-3p | MDM2 | 4193 |
| hsa-mir-29c-3p | MMP2 | 4313 |
| hsa-mir-29c-3p | MMP15 | 4324 |
| hsa-mir-29c-3p | MYCN | 4613 |
| hsa-mir-29c-3p | NASP | 4678 |
| hsa-mir-29c-3p | NEDD9 | 4739 |
| hsa-mir-29c-3p | PDGFRB | 5159 |
| hsa-mir-29c-3p | ENPP2 | 5168 |
| hsa-mir-29c-3p | PER1 | 5187 |
| hsa-mir-29c-3p | PLAG1 | 5324 |
| hsa-mir-29c-3p | PPT1 | 5538 |
| hsa-mir-29c-3p | PPY | 5539 |
| hsa-mir-29c-3p | PRKAB2 | 5565 |
| hsa-mir-29c-3p | PSMA2 | 5683 |
| hsa-mir-29c-3p | PTEN | 5728 |
| hsa-mir-29c-3p | REL | 5966 |
| hsa-mir-29c-3p | REST | 5978 |
| hsa-mir-29c-3p | ABCE1 | 6059 |
| hsa-mir-29c-3p | RPL22 | 6146 |
| hsa-mir-29c-3p | SGK1 | 6446 |
| hsa-mir-29c-3p | SLC16A1 | 6566 |
| hsa-mir-29c-3p | FSCN1 | 6624 |
| hsa-mir-29c-3p | SP1 | 6667 |
| hsa-mir-29c-3p | SPARC | 6678 |
| hsa-mir-29c-3p | SURF2 | 6835 |
| hsa-mir-29c-3p | TARBP1 | 6894 |
| hsa-mir-29c-3p | DYNLT1 | 6993 |
| hsa-mir-29c-3p | TDG | 6996 |
| hsa-mir-29c-3p | TFAP2C | 7022 |
| hsa-mir-29c-3p | TIAM1 | 7074 |
| hsa-mir-29c-3p | TUBB2A | 7280 |
| hsa-mir-29c-3p | VEGFA | 7422 |
| hsa-mir-29c-3p | VHL | 7428 |
| hsa-mir-29c-3p | XK | 7504 |
| hsa-mir-29c-3p | CNBP | 7555 |
| hsa-mir-29c-3p | ZNF45 | 7596 |
| hsa-mir-29c-3p | PTP4A1 | 7803 |
| hsa-mir-29c-3p | BTG2 | 7832 |
| hsa-mir-29c-3p | FZD5 | 7855 |
| hsa-mir-29c-3p | ADAM12 | 8038 |
| hsa-mir-29c-3p | PDHX | 8050 |
| hsa-mir-29c-3p | FZD4 | 8322 |
| hsa-mir-29c-3p | PPM1D | 8493 |
| hsa-mir-29c-3p | FAM193A | 8603 |
| hsa-mir-29c-3p | NUMB | 8650 |
| hsa-mir-29c-3p | CDC23 | 8697 |
| hsa-mir-29c-3p | RIOK3 | 8780 |
| hsa-mir-29c-3p | STK19 | 8859 |
| hsa-mir-29c-3p | PRY | 9081 |
| hsa-mir-29c-3p | COX7A2L | 9167 |
| hsa-mir-29c-3p | SCAF11 | 9169 |
| hsa-mir-29c-3p | DDX21 | 9188 |
| hsa-mir-29c-3p | KLF4 | 9314 |
| hsa-mir-29c-3p | CREB5 | 9586 |
| hsa-mir-29c-3p | MORF4L2 | 9643 |
| hsa-mir-29c-3p | TRAM2 | 9697 |
| hsa-mir-29c-3p | PHACTR2 | 9749 |
| hsa-mir-29c-3p | TESPA1 | 9840 |
| hsa-mir-29c-3p | ZBTB5 | 9925 |
| hsa-mir-29c-3p | AKT3 | 10000 |
| hsa-mir-29c-3p | HUWE1 | 10075 |
| hsa-mir-29c-3p | FEM1B | 10116 |
| hsa-mir-29c-3p | TIMM44 | 10469 |
| hsa-mir-29c-3p | GLRX3 | 10539 |
| hsa-mir-29c-3p | COLEC10 | 10584 |
| hsa-mir-29c-3p | KDM5B | 10765 |
| hsa-mir-29c-3p | SRSF10 | 10772 |
| hsa-mir-29c-3p | MTHFD2 | 10797 |
| hsa-mir-29c-3p | MMP24 | 10893 |
| hsa-mir-29c-3p | MORF4L1 | 10933 |
| hsa-mir-29c-3p | OIP5 | 11339 |
| hsa-mir-29c-3p | SEC31A | 22872 |
| hsa-mir-29c-3p | MAPKBP1 | 23005 |
| hsa-mir-29c-3p | HECW1 | 23072 |
| hsa-mir-29c-3p | KDM6B | 23135 |
| hsa-mir-29c-3p | CEP68 | 23177 |
| hsa-mir-29c-3p | U2SURP | 23350 |
| hsa-mir-29c-3p | KIAA0895 | 23366 |
| hsa-mir-29c-3p | PPP1R13B | 23368 |
| hsa-mir-29c-3p | FRAT2 | 23401 |
| hsa-mir-29c-3p | DICER1 | 23405 |
| hsa-mir-29c-3p | SIRT1 | 23411 |
| hsa-mir-29c-3p | CBX6 | 23466 |
| hsa-mir-29c-3p | BACE1 | 23621 |
| hsa-mir-29c-3p | FJX1 | 24147 |
| hsa-mir-29c-3p | DCAF12 | 25853 |
| hsa-mir-29c-3p | RNF19A | 25897 |
| hsa-mir-29c-3p | WWTR1 | 25937 |
| hsa-mir-29c-3p | CHIC2 | 26511 |
| hsa-mir-29c-3p | BBC3 | 27113 |
| hsa-mir-29c-3p | RAB30 | 27314 |
| hsa-mir-29c-3p | NKIRAS2 | 28511 |
| hsa-mir-29c-3p | SNX24 | 28966 |
| hsa-mir-29c-3p | CD274 | 29126 |
| hsa-mir-29c-3p | PURG | 29942 |
| hsa-mir-29c-3p | SH3GLB1 | 51100 |
| hsa-mir-29c-3p | COMMD2 | 51122 |
| hsa-mir-29c-3p | GOLGA7 | 51125 |
| hsa-mir-29c-3p | BFAR | 51283 |
| hsa-mir-29c-3p | FAM53C | 51307 |
| hsa-mir-29c-3p | RNF138 | 51444 |
| hsa-mir-29c-3p | BCL11A | 53335 |
| hsa-mir-29c-3p | C21orf91 | 54149 |
| hsa-mir-29c-3p | WNT4 | 54361 |
| hsa-mir-29c-3p | OTUD4 | 54726 |
| hsa-mir-29c-3p | TET2 | 54790 |
| hsa-mir-29c-3p | KLHL28 | 54813 |
| hsa-mir-29c-3p | TMEM132A | 54972 |
| hsa-mir-29c-3p | MRM3 | 55178 |
| hsa-mir-29c-3p | ASXL2 | 55252 |
| hsa-mir-29c-3p | SLC30A10 | 55532 |
| hsa-mir-29c-3p | CDV3 | 55573 |
| hsa-mir-29c-3p | UBE2Q1 | 55585 |
| hsa-mir-29c-3p | CAND1 | 55832 |
| hsa-mir-29c-3p | WSB2 | 55884 |
| hsa-mir-29c-3p | RCC2 | 55920 |
| hsa-mir-29c-3p | CDC42SE1 | 56882 |
| hsa-mir-29c-3p | CTNNBIP1 | 56998 |
| hsa-mir-29c-3p | YAE1D1 | 57002 |
| hsa-mir-29c-3p | ZNF286A | 57335 |
| hsa-mir-29c-3p | CNOT6 | 57472 |
| hsa-mir-29c-3p | MKL2 | 57496 |
| hsa-mir-29c-3p | TXNDC16 | 57544 |
| hsa-mir-29c-3p | KIAA1549 | 57670 |
| hsa-mir-29c-3p | RAB40C | 57799 |
| hsa-mir-29c-3p | GNB4 | 59345 |
| hsa-mir-29c-3p | TGIF2 | 60436 |
| hsa-mir-29c-3p | BACH2 | 60468 |
| hsa-mir-29c-3p | P3H1 | 64175 |
| hsa-mir-29c-3p | MMS19 | 64210 |
| hsa-mir-29c-3p | FBRS | 64319 |
| hsa-mir-29c-3p | RFX7 | 64864 |
| hsa-mir-29c-3p | TMEM237 | 65062 |
| hsa-mir-29c-3p | SOWAHC | 65124 |
| hsa-mir-29c-3p | ZBTB10 | 65986 |
| hsa-mir-29c-3p | KCTD15 | 79047 |
| hsa-mir-29c-3p | GTDC1 | 79712 |
| hsa-mir-29c-3p | NAA40 | 79829 |
| hsa-mir-29c-3p | EDC3 | 80153 |
| hsa-mir-29c-3p | CTC1 | 80169 |
| hsa-mir-29c-3p | RAB11FIP1 | 80223 |
| hsa-mir-29c-3p | WDR26 | 80232 |
| hsa-mir-29c-3p | ULBP2 | 80328 |
| hsa-mir-29c-3p | CD276 | 80381 |
| hsa-mir-29c-3p | ZFP91 | 80829 |
| hsa-mir-29c-3p | CSRNP2 | 81566 |
| hsa-mir-29c-3p | ANKRD13C | 81573 |
| hsa-mir-29c-3p | COL21A1 | 81578 |
| hsa-mir-29c-3p | PPP1R14C | 81706 |
| hsa-mir-29c-3p | ISG20L2 | 81875 |
| hsa-mir-29c-3p | SPRTN | 83932 |
| hsa-mir-29c-3p | RHBDD1 | 84236 |
| hsa-mir-29c-3p | RTL6 | 84247 |
| hsa-mir-29c-3p | DOT1L | 84444 |
| hsa-mir-29c-3p | TNRC18 | 84629 |
| hsa-mir-29c-3p | CBX2 | 84733 |
| hsa-mir-29c-3p | KMT5C | 84787 |
| hsa-mir-29c-3p | ADO | 84890 |
| hsa-mir-29c-3p | PHLDB2 | 90102 |
| hsa-mir-29c-3p | OTULIN | 90268 |
| hsa-mir-29c-3p | FMNL3 | 91010 |
| hsa-mir-29c-3p | R3HDM4 | 91300 |
| hsa-mir-29c-3p | ELMSAN1 | 91748 |
| hsa-mir-29c-3p | PIGS | 94005 |
| hsa-mir-29c-3p | C1QTNF6 | 114904 |
| hsa-mir-29c-3p | KLHDC3 | 116138 |
| hsa-mir-29c-3p | AGAP1 | 116987 |
| hsa-mir-29c-3p | IRGQ | 126298 |
| hsa-mir-29c-3p | AMER1 | 139285 |
| hsa-mir-29c-3p | SPIN4 | 139886 |
| hsa-mir-29c-3p | SLC2A14 | 144195 |
| hsa-mir-29c-3p | CCDC117 | 150275 |
| hsa-mir-29c-3p | C4orf26 | 152816 |
| hsa-mir-29c-3p | RAET1L | 154064 |
| hsa-mir-29c-3p | BMT2 | 154743 |
| hsa-mir-29c-3p | TMTC3 | 160418 |
| hsa-mir-29c-3p | ZFPM1 | 161882 |
| hsa-mir-29c-3p | METTL15 | 196074 |
| hsa-mir-29c-3p | TET3 | 200424 |
| hsa-mir-29c-3p | DENND6A | 201627 |
| hsa-mir-29c-3p | TIPRL | 261726 |
| hsa-mir-29c-3p | GAS2L3 | 283431 |
| hsa-mir-29c-3p | FAM102B | 284611 |
| hsa-mir-29c-3p | FAM126B | 285172 |
| hsa-mir-29c-3p | FREM2 | 341640 |
| hsa-mir-29c-3p | GLDN | 342035 |
| hsa-mir-29c-3p | ZNF850 | 342892 |
| hsa-mir-29c-3p | FAM71F2 | 346653 |
| hsa-mir-29c-3p | IRF2BP2 | 359948 |
| hsa-mir-29c-3p | SLC7A5P2 | 387254 |
| hsa-mir-29c-3p | C15orf52 | 388115 |
| hsa-mir-29c-3p | C12orf76 | 400073 |
| hsa-mir-29c-3p | ZBTB34 | 403341 |
| hsa-mir-29c-3p | YY2 | 404281 |
| hsa-mir-29c-3p | PRY2 | 442862 |
| hsa-mir-29c-3p | TRIM72 | 493829 |
